# Associations of Organophosphate Pesticide Metabolite Mixtures with Epigenetic Age Acceleration

**DOI:** 10.3390/jox16060182

**Published:** 2026-09-25

**Authors:** Taújay Davis, Emmanuel Obeng-Gyasi

**Affiliations:** 1 Department of Built Environment, North Carolina A&T State University, Greensboro, NC 27411, USA; 2 Environmental Health and Disease Laboratory, North Carolina A&T State University, Greensboro, NC 27411, USA

**Keywords:** organophosphate pesticides, dialkylphosphates, epigenetic aging, epigenetic clocks, Bayesian kernel machine regression, environmental mixtures, NHANES

## Abstract

Organophosphate (OP) pesticide exposure occurs as complex mixtures, yet joint associations with DNA methylation-derived aging measures remain poorly characterized. We examined six urinary dialkylphosphate (DAP) metabolites in relation to epigenetic age acceleration (EAA) among 427 adults aged ≥50 years in the 1999–2002 National Health and Nutrition Examination Survey. EAA was derived from the Horvath, Hannum, PhenoAge, GrimAge, and GrimAge2 clocks. Metabolites were measured in a single spot urine specimen, so exposure reflects recent rather than cumulative exposure. Survey-weighted multivariable linear regression estimated conventional associations, and unweighted Bayesian kernel machine regression (BKMR) characterized potentially nonlinear mixture associations. No DAP–clock association reached nominal significance or survived Benjamini–Hochberg false-discovery-rate correction across 30 tests. BKMR suggested heterogeneous, clock-dependent patterns: posterior mean mixture–response estimates increased at higher quantiles for Horvath, Hannum, and PhenoAge but not for GrimAge or GrimAge2. DEDTP had the highest posterior inclusion probability in each primary model, though magnitudes were modest for the GrimAge clocks. No association survived FDR correction after leukocyte-composition adjustment. Posterior support for pairwise interactions was weak, and credible intervals were wide. Given the cross-sectional design and discordant directions across clocks, these exploratory findings do not establish that DAP exposure accelerates biological aging and warrant longitudinal confirmation.

## 1. Introduction

Organophosphates (OPs) are common chemicals used for pesticide treatments, but dietary, residential and occupational environment exposures to these OPs interrupt the nervous system through the inhibition of acetylcholinesterase. Parent OPs such as disulfoton, phorate, and malathion induce this toxicity and are metabolized to non-specific urinary dialkylphosphate (DAP) metabolites. Diet is a recognized route of OP exposure, and studies have reported associations between consumption of fruits and vegetables treated with OPs and higher urinary metabolite concentrations. Urinary samples containing DAP metabolites reflect exposure and may be identified via Gas Chromatography-Linked Tandem Mass Spectrometry (GC-MS/MS) [1]. However, urinary DAPs may also be signifiers of preformed DAP metabolites in the environment and therefore do not uniquely identify specific parent pesticides and their respective toxicological potencies. These DAP biomarkers consist of diethyl phosphate (DEP), diethyl thiophosphate (DETP), diethyl dithiophosphate (DEDTP), dimethyl phosphate (DMP), dimethyl thiophosphate (DMTP), and dimethyl dithiophosphate (DMDTP). Exposure to the parent OP compounds has been associated with cardiometabolic dysregulation and with neurotoxic and immunological effects; the DAP metabolites measured in urine are excretion products of that exposure and are not themselves the agents of these effects [2,3]. Organophosphate exposure has been associated with oxidative stress, inflammation, and alterations in DNA methylation, providing biological plausibility for investigating whether OP exposure may also be associated with DNA methylation-based measures of aging. However, evidence directly linking OP pesticide exposure to epigenetic age acceleration remains limited, particularly for simultaneous exposure to multiple OP metabolites. Thus, epigenetic age acceleration is considered here as an aging-related molecular phenotype rather than as evidence that OP exposure directly accelerates the biological aging process. Exposure to parent organophosphate pesticides from multiple sources is detrimental to human health, and the relationship between urinary metabolite mixtures and biological aging has yet to be thoroughly examined. Urinary DAP concentrations reflect recent exposure and are not a measure of cumulative lifetime exposure.

### 1.1. Epigenetic Aging and Acceleration

Epigenetic aging refers to age-related variation in DNA methylation patterns that can be summarized using epigenetic clock algorithms. These algorithms estimate DNA methylation-derived age or related aging phenotypes from selected CpG sites, although the biological information represented by individual clocks differs according to their training targets and CpG composition [4,5]. Epigenetic age acceleration (EAA) is commonly operationalized as the residual from regressing DNA methylation-predicted age on chronological age. A positive residual therefore indicates that an individual’s clock-estimated age is older than expected for their chronological age, whereas a negative residual indicates a younger-than-expected clock estimate. Importantly, EAA should not be interpreted as a direct measurement of organismal aging, organ failure, or the rate of biological aging; rather, it is a clock-specific biomarker that has been associated with aging-related phenotypes, morbidity, and mortality. The biological interpretation of EAA is also clock-dependent. First-generation clocks such as Horvath and Hannum were optimized primarily to predict chronological age, whereas later-generation clocks such as PhenoAge and GrimAge incorporated phenotypic or mortality-related information into their development [5]. Consequently, associations with an environmental exposure need not be identical across clocks, and discordant findings should not necessarily be interpreted as contradictory evidence [6]. Rather, they may reflect differences in the biological domains captured by the respective algorithms. Positive age acceleration has been associated with an elevated risk of disease and with impairment of cognitive function [7,8]. Together, these epigenetic clocks have been used to evaluate whether xenobiotic exposures such as chemicals inhaled from tobacco smoking or organophosphates from pesticides in occupational and environmental settings are associated with DNA methylation-based measures of age acceleration, which have in turn been associated with heightened risks for chronic disease, morbidity, and mortality [9,10]. Previous NHANES studies have examined organophosphate biomarkers and epigenetic aging using conventional, nonlinear, and exposome-wide approaches [11,12]. A recent analysis of the same NHANES 1999–2002 DNA methylation resource examined urinary organophosphate metabolites in relation to DNA methylation aging measures and heart disease mortality among 502 adults aged 50 years and older [3]. That analysis evaluated metabolites one at a time as candidate mediators of a mortality endpoint, whereas the present study treats the six-metabolite dialkylphosphate panel as a joint exposure and epigenetic age acceleration as the outcome. The present study extends this literature by evaluating six urinary dialkylphosphate metabolites simultaneously and applying both multivariable regression and Bayesian kernel machine regression to characterize joint exposure associations, nonlinear exposure–response relationships, relative metabolite importance, and potential interactions across five epigenetic clocks.

### 1.2. Organophosphate Metabolism and Influence on Epigenetic Aging

Emerging epidemiologic evidence suggests that organophosphate exposure may be associated with DNA methylation-based aging measures; however, the evidence remains limited and does not establish that OP exposure causes accelerated biological aging. In one such investigation, which evaluated four dialkylphosphate metabolites, DEP carried the largest weight in a weighted quantile sum model of combined exposure, ahead of DMP, DETP, and DMTP [11]. Circulating C-reactive protein was reported to partially mediate the association between those four metabolites and an algorithm-based measure of biological aging acceleration, with mediated proportions between 22% and 33% [11]. Oxidative stress and inflammation associated with organophosphate exposure have also been proposed as pathways through which DNA methylation patterns may be altered [13,14]. The rationale for examining combined OP exposure is further supported by evidence that individual organophosphate exposures may be associated with molecular and physiological processes relevant to aging [11]. A systematic review and meta-analysis of pesticide exposure and biological aging found only four studies of epigenetic clocks and reported no significant pooled association with Hannum clock acceleration [15], indicating that the epigenetic-clock evidence base for pesticides remains small and that pooled estimates to date have been compatible with the null. However, most previous studies have evaluated individual pesticides or metabolites rather than simultaneous exposure to multiple compounds. Because human exposure occurs within complex environmental mixtures, evaluating DAP metabolites jointly may better characterize exposure patterns that are not captured by single-pollutant models. Accordingly, investigation of OP mixtures in relation to multiple DNA methylation-derived aging measures may provide insight into potentially heterogeneous and nonlinear exposure–response patterns. The objectives of this study were to evaluate associations between individual and combined DAP metabolites and epigenetic age acceleration across five epigenetic clocks, characterize potentially nonlinear mixture–response patterns, and identify metabolites with greater relative posterior importance within the fitted mixture models while adjusting for chronological age, sex, race/ethnicity, education, household income, BMI, smoking status, and urinary creatinine.

Accordingly, the premise of the present study is not that urinary DAP concentrations necessarily cause accelerated biological aging, but that DAP exposure patterns may be associated with variation in established DNA methylation-derived age-acceleration measures. We therefore examined whether individual and combined DAP metabolite concentrations were associated with clock-specific EAA measures and whether flexible mixture modeling identified exposure–response patterns not captured by conventional linear models. Given differences in clock construction and the limited prior evidence for OP mixtures, analyses across the five clocks were considered complementary and exploratory rather than tests of a single uniform biological-aging construct.

## 2. Materials and Methods

### 2.1. Study Population

The National Health and Nutrition Examination Survey (NHANES) produced public data on urinary OP metabolites along with measurements of epigenetic aging based on altered DNA methylation [16]. We conducted a cross-sectional analysis of NHANES data collected during 1999–2002. Eligible participants were adults aged 50 years or older with available DNA methylation biomarkers, urinary DAP measurements, and complete covariate information. Participants were excluded if they were missing any required covariate, including those with nonresponse household-income codes or indeterminate smoking status (Section 2.5.5). Chronological age is top-coded at 85 years in the public-use files, and this top-coding was retained. The analytic sample is the intersection of the NHANES DNA methylation subsample and the urinary pesticide subsample among adults aged 50 years and older; participant counts at each stage of assembly are shown in Figure 1, and sample characteristics are summarized in Table 1. All participants within this study issued informed consent, and the NHANES collection of data received IRB approval.

### 2.2. Exposure Assessment: Organophosphate Metabolites

The exposure assessment embedded in this research observed the organophosphate metabolites found in the urinary samples of each adult represented in the final analytic sample. The DAP panel comprised six urinary dialkylphosphate metabolites: dimethyl phosphate (DMP), diethyl phosphate (DEP), dimethyl thiophosphate (DMTP), diethyl thiophosphate (DETP), dimethyl dithiophosphate (DMDTP), and diethyl dithiophosphate (DEDTP). Urinary DAP concentrations were measured by the Division of Laboratory Sciences, National Center for Environmental Health (NCEH), Centers for Disease Control and Prevention (CDC), using isotope-dilution gas chromatography-tandem mass spectrometry (GC-MS/MS); specimen handling, sample preparation, instrumental conditions, and quality assurance/quality control (QA/QC) procedures are described below. The six DAP metabolites were entered simultaneously in every exposure–outcome model, with metabolite-specific estimates reported alongside the joint mixture summaries. Biomarker z-score standardization was performed for conventional, linear regression and BKMR modeling, while creatinine normalization was implemented on DAP concentrations for descriptive reporting. Cycle-specific analytical Limit of Detection (LOD) values were retrieved from the NHANES laboratory documentation [17,18]. The values below the LOD were handled using NHANES convention as values <LOD were replaced with LOD/√2. With respect to the final analytic sample, detection frequencies and percentages below the LOD were calculated using the applicable cycle-specific LOD and are reported in Supplementary Table S3.

*Specimen collection and handling.* Spot urine specimens were collected in the NHANES mobile examination center (MEC) during the scheduled examination session rather than as first-morning voids. Participants were instructed to wash their hands, to keep the collection cup and cap from touching the body, clothing, or other surfaces, and to close the container promptly to limit exposure to air [19]. For participants selected into the organophosphate subsample, a 10 mL aliquot was poured into a dedicated 14 mL snap-cap vessel, frozen in MEC freezers maintained at −18 to −23 °C, and shipped weekly on dry ice to the CDC NCEH laboratory [19]. Specimens were stored at −20 °C until analysis, and residual urine was returned to storage at −70 °C after aliquoting [20].

*Sample pre-treatment and addition of internal standards.* Specimens, reagents, and standards were brought to room temperature, and a 2 mL urine aliquot was spiked with 10 µL of a mixed isotopically labeled internal standard solution containing DMP-d6, DEP-d10, DMTP-d6, DMDTP-d6, DETP-d10, and DEDTP-^13^C4 (isotopic purity >99%; Cambridge Isotope Laboratories, Andover, MA, USA), giving a urinary concentration of 25 µg/L for each labeled analyte [20,21]. Because a stable isotope-labeled analogue of every metabolite was added before any sample manipulation, each internal standard corrected for sample-specific losses during extraction and derivatization and for variation in instrument response. Water was then removed by overnight lyophilization (Labconco, Kansas City, MO, USA) using a programmed cycle of freezing at −34 °C, drying under vacuum (25.5 mTorr), and stepwise warming to 20 °C. Lyophilization was used in place of liquid–liquid extraction, solid-phase extraction, or azeotropic distillation because the DAPs are highly polar, with low pKa values, and those approaches gave low or concentration-dependent recoveries [20,21].

*Extraction, purification, and derivatization.* The dried residue was extracted with 2 mL of acetonitrile and 2 mL of diethyl ether with vortex mixing, and the supernatant was decanted from the undissolved residue; the residue was rinsed with a further 1 mL of acetonitrile, and the combined extract was concentrated to approximately 1 mL under nitrogen (TurboVap LV, Zymark, Hopkinton, MA, USA; 30 °C, 10 psi). The DAPs were derivatized to their chloropropyl phosphate esters by adding 50 µL of 1-chloro-3-iodopropane in the presence of potassium carbonate and heating at 60 °C for 3 h. The supernatant was transferred to a clean tube, evaporated to dryness, reconstituted in 75 µL of toluene, and transferred to autosampler vials that were kept refrigerated until analysis [20,21].

*Instrumental analysis and quantification.* Extracts (1 µL) were injected in splitless mode onto a TraceGC gas chromatograph (ThermoQuest, San Jose, CA, USA) fitted with a deactivated fused-silica guard column and a DB-5MS capillary column (30 m × 0.25 mm i.d., 0.25 µm film; J&W, Folsom, CA, USA). Injector and transfer-line temperatures were 250 °C, and the oven was held at 80 °C for 2 min, ramped at 17 °C/min to 250 °C, and held for 2 min. Detection used a Finnigan TSQ-7000 triple quadrupole mass spectrometer (ThermoFinnigan, San Jose, CA, USA) operated with positive-ion chemical ionization (methane reagent gas) and argon collision-induced dissociation in multiple reaction monitoring mode. For each analyte and internal standard, the protonated pseudomolecular ion containing ^35^Cl served as the quantification precursor and the corresponding ^37^Cl ion as the confirmation precursor. Positive identification required the expected retention time, both precursor ions, their characteristic product ions, and a product–ion ratio within a predefined range. Concentrations were quantified by isotope dilution from seven-point calibration curves prepared in blank urine and processed with each analytical run, constructed by linear regression of the native-to-labeled peak-area ratio on standard concentration. The lowest standard was at or below the LOD, the linear range extended from the LOD to 50 µg/L, and calibration curves were required to have R^2^ > 0.97; specimens exceeding the calibration range were reanalyzed using a smaller aliquot [20].

*Quality assurance and quality control.* Each analytical batch contained a reagent blank, one low and one high QC sample, seven calibration standards, and up to 36 unknown specimens [20]. QC materials were prepared from urine pooled from 430 donors, diluted 1:1 with water, and filtered (0.2 µm); one pool was left unspiked for use as a blank matrix, and the others were spiked to approximately 8 µg/L (low pool; 4 µg/L for DMP and 0.6 µg/L for DEDTP) and 20 µg/L (high pool; 10 µg/L for DMP and 1.3 µg/L for DEDTP). Control limits at the 95% and 99% levels were established from at least 20 consecutive characterization runs of each pool. A run was accepted only when the reagent blank was free of target analytes and QC results satisfied the Westgard multirules (1-3s, 2-2s, R-4s, and 10-x) [20,22]; data from out-of-control runs were not reported, and analyses were suspended until the cause was identified and corrected. Independently prepared calibration verification standards were analyzed at least every 6 months and after any substantive change to the method or instrumentation, and results for an analyte were not reported if an interference with its internal standard persisted on reanalysis. The LOD for each analyte was calculated as 3s0, where s0 is the standard deviation at zero concentration estimated by linear regression of the standard deviation on the concentration for the four lowest standards, and was verified by analysis of urine spiked at similar concentrations [20]. The method has a reported precision of 7–14% (coefficient of variation) [21]. During 2001–2002 analyses (April to December 2001; 70 low and 70 high QC results per analyte), between-run coefficients of variation for the QC pools ranged from 11.3% to 19.7% across the six metabolites [20]. Specimens from the 1999–2000 cycle were analyzed by the same CDC laboratory using isotope-dilution GC-MS/MS with stable isotope-labeled internal standards [23]. Cycle-specific LODs are provided in Supplementary Table S3.

### 2.3. Outcome Assessment: Epigenetic Aging

To quantify epigenetic aging, DNA methylation assays, verified by quality control (QC), were used. Different epigenetic clocks were utilized to further assess biological aging in each individual from the final analytic dataset: Horvath, Hannum, PhenoAge, GrimAge, and GrimAge2 [24,25,26,27,28]. These five clocks were selected to represent both first-generation predictors of chronological age and subsequent generations trained on phenotypic and mortality factors. Though GrimAge and GrimAge2 are highly correlated, both were retained in this study to evaluate how consistent findings were between the original and updated algorithms. These algorithms produce a biological age after quantifying methylation levels at the CpG sites incorporated into each epigenetic clock. To measure the acceleration of epigenetic aging, the residuals from regressing DNA methylation-predicted age on chronological age were computed separately for each clock. DNA methylation was measured in purified whole-blood DNA by NCHS using the Illumina MethylationEPIC BeadChip (Illumina, Inc., San Diego, CA, USA). NCHS processed the raw intensity (IDAT) files first, then applied background correction and normalization, followed by quality-control screening of samples and probes, and finally, computed the epigenetic age and related DNA methylation biomarkers from the processed data. Only these derived biomarker variables were released publicly; CpG-level methylation data and the underlying IDAT files were not released in the public-use files [29]. These derived biomarker measures were used for the present study, and each epigenetic age-acceleration outcome was modeled as a continuous variable. NCHS also released estimated leukocyte cell-type proportions derived from the same array data. These proportions were not incorporated into the released clock estimates and were not adjusted for in the primary regression or BKMR models, but they were included in a sensitivity analysis of the conventional regression models (Section 2.5.3; Supplementary Table S4). The resulting residuals were interpreted as clock-specific age-acceleration measures rather than direct estimates of the rate of biological aging. Positive values indicate an older-than-expected DNAm age conditional on chronological age, and negative values indicate a younger-than-expected DNAm age. Because the clocks differ in their training objectives and biological composition, estimates were interpreted separately for each clock rather than as interchangeable measures of a single latent aging process.

### 2.4. Covariates

For this study, the covariates included in the final analytic models were chronological age, sex, race/ethnicity, education, household income, body mass index (BMI), smoking status, and urinary creatinine. Sex and race/ethnicity were included as demographic factors, while education and household income were included to account for socioeconomic differences. BMI was included as a health-related covariate, and urinary creatinine was included to account for variation in urine dilution. Chronological age was retained in the study as a needed factor in EAA estimation, and smoking status was incorporated into the covariates as it has been established as having significant associations with EAA [9].

### 2.5. Statistical Analysis

#### 2.5.1. Descriptive Statistics

Participant characteristics were summarized using survey-weighted means and standard errors for continuous variables as well as weighted percentages for categorical variables. Because the BKMR models are unweighted and prior analyses of organophosphates and epigenetic aging have reported unweighted descriptive statistics, unweighted means and standard deviations for continuous variables and unweighted counts and percentages for categorical variables are reported alongside the survey-weighted estimates in Supplementary Table S5. The urinary DAP concentrations were reported using both unadjusted (µg/L) and creatinine-adjusted (µg/g creatinine) values.

#### 2.5.2. Correlation Analysis

Pairwise Spearman rank correlations were calculated using Stata 19 (StataCorp LLC, College Station, TX, USA) and evaluated in the final analytic sample (*n* = 427) for every pair among the six DAP metabolites.

#### 2.5.3. Multivariable Linear Regression

Multivariable linear regression was performed for DAP metabolites and epigenetic age acceleration to produce estimations about their individual associations. Accelerated epigenetic aging was identified by calculating the residuals from linear regression models regressing each epigenetic clock age (HorvathAge, HannumAge, PhenoAge, GrimAge, GrimAge2) on the respective chronological age of the sample’s participants [30]. The model produced in this research simultaneously assessed the exposure of the mixture of metabolites within the DAP panel, and each β coefficient was mutually adjusted for the other five metabolites. The adjustments considered for the model included the covariates previously listed: Chronological Age, Sex, Ethnicity, Education, Income, BMI, Smoking Status, and Urinary Creatinine. The outputs yield β per 1-SD coefficients and 95% confidence intervals. NHANES strata, primary sampling units (PSUs), and survey weights were incorporated into all regression models. Categorical covariates were entered as indicator variables (sex, 2 levels; race/ethnicity, 5; education, 5; household income, 12; smoking status, 3), with the first level of each serving as the referent. Benjamini–Hochberg false-discovery-rate correction was applied across the 30 primary DAP-clock tests. Because variance inflation factors are a design-matrix diagnostic and are not defined under survey estimation, VIFs were computed from the corresponding unweighted ordinary least squares fit of the same model. Two sensitivity analyses of the conventional regression models were conducted. In the first, the NCHS-derived leukocyte cell-type proportions (CD8+ T cells, CD4+ T cells, natural killer cells, B cells, and monocytes, with neutrophils omitted as the referent composition) were added to the covariate set. In the second, household income was collapsed to a binary indicator of below versus at or above $20,000, which also resolves the NHANES income code denoting only that household income exceeded $20,000. Benjamini–Hochberg correction was applied within each sensitivity model across its own 30 tests. Results are reported in Supplementary Table S4.

#### 2.5.4. Mixture Analysis (BKMR)

To provide estimations of the combined association of the six metabolites within the DAP panel with accelerated epigenetic aging, evaluations for nonlinear exposure associations and response patterns, and the relative importance of individual exposures within the mixture, Bayesian Kernel Machine Regression was applied to the results using 5000 iterations for each epigenetic clock analysis [31]. The model incorporated an estimation of Markov Chain Monte Carlo (MCMC) sampling and consisted of two components: the Kernel function for the exposure mixture and the Covariate-adjusted framework. A single MCMC chain was run for each clock-specific model. Convergence was assessed by visual inspection of trace plots and by comparing posterior inclusion probabilities and overall mixture–response summaries across chain lengths and random seeds; formal multi-chain diagnostics such as the Gelman–Rubin statistic were therefore not computed. BKMR analyses were unweighted, and the outputs included the overall mixture–response function, exposure–response functions, and posterior inclusion probabilities (PIPs), which were used to characterize the relative posterior importance of individual metabolites within each clock-specific mixture–outcome model. Inference from the BKMR models was based on the full posterior distributions rather than null-hypothesis significance testing. The overall-mixture and single-variable summaries utilized the resulting posterior mean and standard deviations, which were factored into the 95% credible interval calculation as the posterior mean ± 1.96 × posterior standard deviation. Posterior estimates and uncertainty measures from the BKMR prediction functions formulated the univariate exposure–response functions. As a sensitivity analysis, all BKMR models were rerun using 20,000 MCMC iterations with the same covariate, variable-selection, exposure matrix, and knot structure specifications as the primary 5000-iteration models. Iterations 2501–5000 were used for the primary posterior summaries and iterations 10,001–20,000 for the sensitivity runs. An additional, independent 20,000-iteration GrimAge2 model was fitted because the initial GrimAge2 sensitivity analysis displayed greater variability in component ranking across MCMC chain lengths. Full BKMR and sensitivity details along with respective trace plots are provided in Supplementary Methods S1 and Figures S6 and S7.

#### 2.5.5. Missing Data Handling

Participants without complete covariate data were excluded, yielding a complete-case analytic sample of 427. This exclusion includes individuals with nonresponse income codes and an indeterminate smoking status, and may reduce precision and affect the composition of the analytic sample. Sample characteristics are summarized in Table 1.

### 2.6. Survey Design and Software

Descriptive and conventional regression analyses were design-based and used the NHANES strata, primary sampling units, and the four-year DNA methylation subsample weight. The analytic sample is the intersection of the DNA methylation subsample and the pesticide subsample, and no official weight is published for that intersection; the available NHANES weight was therefore used as an approximation. Stata 19 (StataCorp LLC, College Station, TX, USA) was used for the descriptive statistics, Spearman correlations, and linear regression, with a significance threshold of α = 0.05 for frequentist models. Then, the BKMR analyses were computed using R software (version 4.5; R Foundation for Statistical Computing, Vienna, Austria) with the bkmr package (version 0.2.2). The Bayesian Kernel Machine Regression mixture models were not survey-weighted and do not incorporate the NHANES sampling weights, strata, or primary sampling units; these results are therefore unweighted.

## 3. Results

### 3.1. Descriptive Statistics Overview of Study Participants

The demographic information and participant statistics are outlined in Table 1. The descriptive statistics showcased the variation in covariates: sex, ethnicity, BMI, income, education level, and creatinine levels. The average age among all participants was 60.78 years among the subpopulation, consisting of 47.02% men and 52.98% women. Ethnic groups comprised Non-Hispanic White (80.61%), Non-Hispanic Black (6.87%), Mexican American (3.27%), Other Race—Including Multi-Racial (4.92%), and Other Hispanic (4.33%). The mean BMI was 28.79 kg/m^2^, and the mean creatinine was 106.73 mg/dL. As for the exposure variables—DMP, DEP, DMTP, DETP, DMDTP, DEDTP—in the study subpopulation, the averages (µg/L) were 2.84, 2.75, 9.50, 1.10, 2.74, and 0.21, respectively. Upon creatinine adjustment, these values became 3.22, 2.51, 9.47, 1.32, 2.92, and 0.36, respectively. The education levels ranged among the following groups: Less Than 9th Grade (6.79%), 9–12th Grade (15.80%), High School Graduate/GED or Equivalent (24.37%), Some College or AA degree (26.32%), College Graduate or Above (26.73%). The distribution of income groups and their respective percentages pertaining to the study subpopulation are also displayed in Table 1 as categories ranging from $0 to $75,000 and Over. Lastly, the different categories of smokers were split among Never (40.88%), Former (40.77%), and Current (18.35%). Unweighted descriptive statistics for the same variables are provided in Supplementary Table S5. The weighted and unweighted distributions differ appreciably for race/ethnicity and, to a lesser extent, for age and education, which follows from the sampling design of the DNA methylation subsample described in Section 4.3. Unweighted, the analytic sample was 47.31% non-Hispanic White, 24.36% Mexican American, 18.27% non-Hispanic Black, 5.39% other Hispanic, and 4.68% other race including multi-racial, with a mean age of 62.92 years (SD 10.41) compared with a survey-weighted mean of 60.78 years. The unweighted distribution is the one represented in the BKMR models.

### 3.2. Correlation Analysis for Exposure Variables

The Spearman correlation analysis for the DAP panel is displayed in Figure 2. All pairwise correlations among the six DAP metabolites were weak, with the exception of three pairs: DMP-DMTP, DETP-DEDTP, and DMTP-DMDTP, which had a moderate correlation.

### 3.3. Multivariable Linear Regression: Accelerated Epigenetic Aging

Stata regression results shown in Figure 3 display the coefficient plot of DAP panel exposure versus accelerated epigenetic aging among epigenetic clocks Horvath, Hannum, PhenoAge, GrimAge, and GrimAge2 after adjusting for covariates: chronological age, sex, BMI, education level, income, creatinine, smoking status, and ethnicity. Each estimate is reported with its 95% confidence interval rather than being interpreted against a significance threshold alone. Positive and negative coefficients indicate estimated positive and inverse associations, respectively. DMP consistently had positive coefficients across all clocks. In contrast, DEP outcomes consisted of all negative coefficients. DMTP had positive coefficients for HorvathAge and HannumAge while exhibiting negative coefficients for second-generation epigenetic clocks. Positive coefficients under HorvathAge, HannumAge, and PhenoAge were observed for DETP, while it had negative coefficients for both GrimAge and GrimAge2. All clocks except for PhenoAge had positive coefficients when assessing the associations between DMDTP and EAA. Conversely, all clocks except HorvathAge had negative coefficients for the associations involving DEDTP. Intervals that cross the β = 0 vertical line are consistent with the null. All 95% confidence intervals for the metabolites among all epigenetic clocks overlapped β = 0 with coefficient plots surrounding the center; no test reached nominal significance, and none survived Benjamini–Hochberg FDR correction (smallest *p* = 0.054). Therefore, the entirety of the conventional linear regression analyses did not provide statistically significant associations between dialkylphosphate exposure and alteration to epigenetic aging measures. In addition, VIFs accompanying the regression data are presented in Table S1 and ranged from 1.11 to 1.56 among all DAP metabolites. Neither sensitivity analysis changed this conclusion (Supplementary Table S4). After additional adjustment for leukocyte cell-type proportions, no DAP–clock association survived FDR correction (smallest q = 0.264), although two associations reached nominal significance that were not nominally significant in the primary models (DEP with GrimAge, β = 0.37, *p* = 0.009; DMTP with GrimAge2, β = −0.60, *p* = 0.045). Collapsing household income to below versus at or above $20,000 likewise left no association significant after correction (smallest q = 0.676).

**Figure 3:**
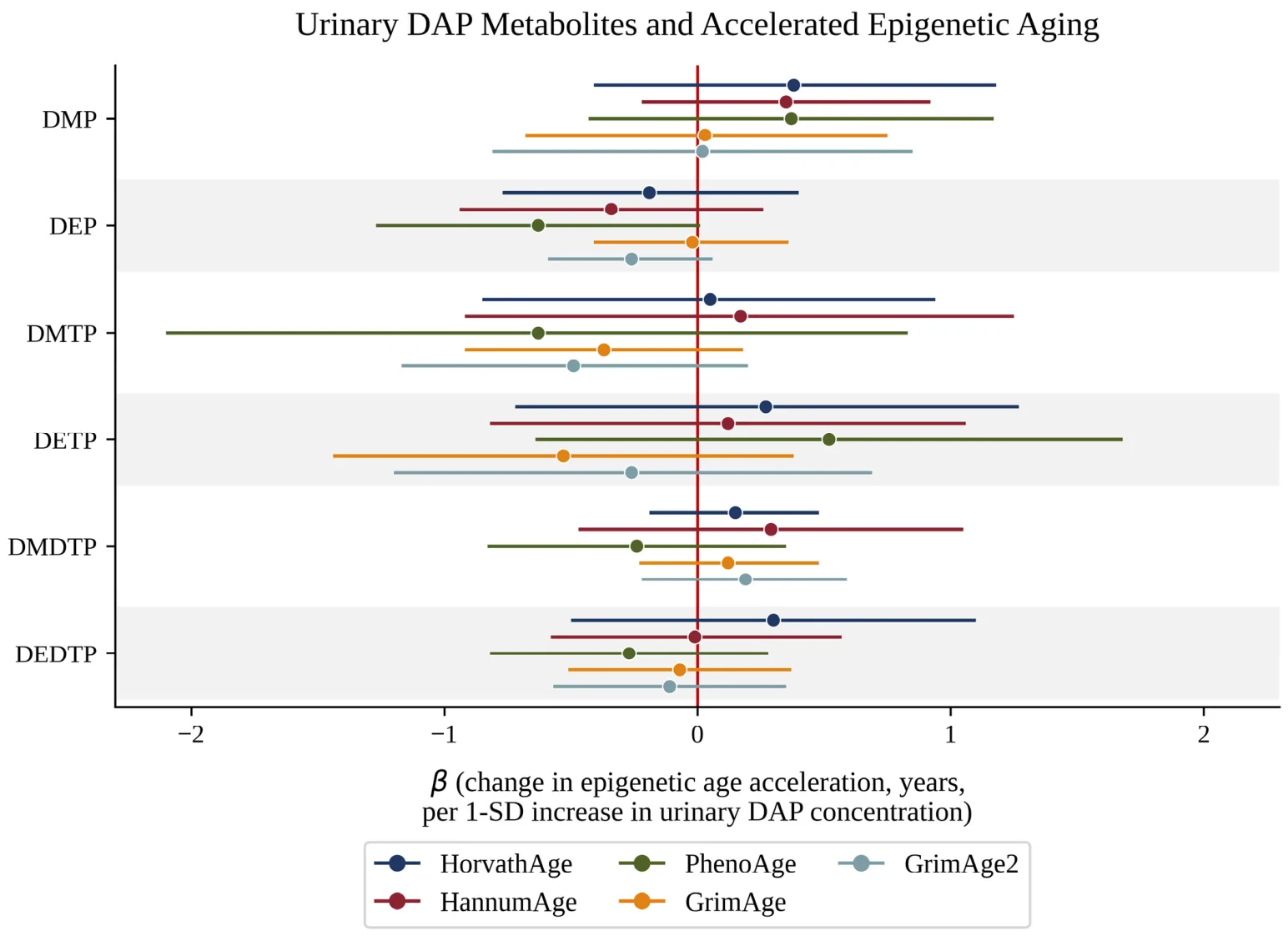
Coefficient plot for multivariable linear regression among epigenetic clocks: DAP panel exposure vs. accelerated epigenetic aging. Points are survey-weighted β coefficients, and horizontal bars are 95% confidence intervals, expressed per one-standard-deviation increase in the corresponding standardized urinary DAP concentration; each coefficient is mutually adjusted for the other five DAP metabolites and for chronological age, sex, race/ethnicity, education, household income, BMI, smoking status, and urinary creatinine. Numeric values are given in Table 2. DEP—diethylphosphate, DMP—dimethylphosphate, DMTP—dimethylthiophosphate, DETP—diethylthiophosphate, DMDTP—dimethyldithiophosphate, DEDTP—diethyldithiophosphate.

**Table 2:** Regression data for multivariable linear regression among epigenetic clocks using standardized values: DAP Panel exposure vs. accelerated epigenetic aging.

**HorvathAge**				
**DAP Metabolite**	**β (SE)**	**95% CI**	***p*-value**	**BH-FDR q-value**
DMP	0.38 (0.39)	−0.41 to 1.18	0.330	0.785
DEP	−0.19 (0.29)	−0.77 to 0.40	0.519	0.820
DMTP	0.05 (0.44)	−0.85 to 0.94	0.917	0.974
DETP	0.27 (0.49)	−0.72 to 1.27	0.580	0.829
DMDTP	0.15 (0.16)	−0.19 to 0.48	0.375	0.785
DEDTP	0.30 (0.39)	−0.50 to 1.10	0.445	0.785
**HannumAge**				
DMP	0.35 (0.28)	−0.22 to 0.92	0.223	0.785
DEP	−0.34 (0.29)	−0.94 to 0.26	0.258	0.785
DMTP	0.17 (0.53)	−0.92 to 1.25	0.751	0.939
DETP	0.12 (0.46)	−0.82 to 1.06	0.795	0.954
DMDTP	0.29 (0.37)	−0.47 to 1.05	0.443	0.785
DEDTP	−0.01 (0.28)	−0.58 to 0.57	0.974	0.974
**PhenoAge**				
DMP	0.37 (0.39)	−0.43 to 1.17	0.348	0.785
DEP	−0.63 (0.31)	−1.27 to 0.01	0.054	0.785
DMTP	−0.63 (0.72)	−2.10 to 0.83	0.386	0.785
DETP	0.52 (0.56)	−0.64 to 1.68	0.365	0.785
DMDTP	−0.24 (0.29)	−0.83 to 0.35	0.408	0.785
DEDTP	−0.27 (0.27)	−0.82 to 0.28	0.323	0.785
**GrimAge**				
DMP	0.03 (0.35)	−0.68 to 0.75	0.923	0.974
DEP	−0.02 (0.19)	−0.41 to 0.36	0.902	0.974
DMTP	−0.37 (0.27)	−0.92 to 0.18	0.182	0.785
DETP	−0.53 (0.44)	−1.44 to 0.38	0.243	0.785
DMDTP	0.12 (0.17)	−0.23 to 0.48	0.490	0.816
DEDTP	−0.07 (0.22)	−0.51 to 0.37	0.739	0.939
**GrimAge2**				
DMP	0.02 (0.41)	−0.81 to 0.85	0.955	0.974
DEP	−0.26 (0.16)	−0.59 to 0.06	0.109	0.785
DMTP	−0.49 (0.34)	−1.17 to 0.20	0.159	0.785
DETP	−0.26 (0.46)	−1.20 to 0.69	0.580	0.829
DMDTP	0.19 (0.20)	−0.22 to 0.59	0.352	0.785
DEDTP	−0.11 (0.22)	−0.57 to 0.35	0.621	0.847

DEP—diethylphosphate, DMP—dimethylphosphate, DMTP—dimethylthiophosphate, DETP—diethylthiophosphate, DMDTP—dimethyldithiophosphate, DEDTP—diethyldithiophosphate.

### 3.4. Bayesian Kernel Machine Regression Analysis

#### 3.4.1. Overall Mixture–Response Association

The estimated mixture–response association between DAP concentrations and epigenetic age acceleration is illustrated in Figure 4. Among the epigenetic clocks, the mixture–response outcomes varied in both direction and magnitude in relation to the association as the exposure level increased. For HorvathAge, HannumAge, and PhenoAge, the response estimate increased as the exposure quantile increased. In contrast, the estimated response was flat to decreasing across mixture quantiles for GrimAge and decreased above the median for GrimAge2. In the 20,000-iteration sensitivity analyses, the overall mixture–response patterns were consistent with the primary models for HorvathAge, HannumAge, PhenoAge, and GrimAge, and DEDTP retained the highest posterior inclusion probability within each of these four clocks. PIP magnitudes were most stable for HannumAge and shifted more substantially for HorvathAge, PhenoAge, and GrimAge (Table S2). GrimAge2 demonstrated greater variability in component ranking and posterior mean direction across MCMC specifications, with DEDTP and DMDTP effectively indistinguishable in the longer run; the corresponding mixture estimates nevertheless remained close to null with substantial posterior uncertainty. An additional independent GrimAge2 model initialized with a different random seed likewise supported a weak and uncertain mixture signal (Supplementary Methods S1).

#### 3.4.2. Posterior Inclusion Probabilities Analysis

The Posterior Inclusion Probabilities estimates from the Bayesian Kernel Machine Regression analysis for each organophosphate across all epigenetic clocks in this study are included in Table 3 and Figure 5. PIPs were compared across metabolites within each clock-specific model rather than against any fixed threshold. The PIPs observed here were diffuse and were interpreted as relative, graded evidence of importance rather than as definitive selection of individual metabolites. In the primary models, DEDTP had the highest PIP within every clock, with the strongest posterior support in the HannumAge outcome then HorvathAge, a moderate PIP value in PhenoAge, and the weakest magnitudes in GrimAge then GrimAge2. The absolute magnitudes for GrimAge and GrimAge2 were low, and differences among metabolites within those two models were small.

#### 3.4.3. Secondary BKMR Analyses

The suite of BKMR figures besides the overall mixture response is shown in Supplementary Figures S1–S7. Heterogeneity and nonlinear posterior mean patterns were examined for several relationships between metabolites and EAA among the epigenetic clocks within the univariate and bivariate exposure–response functions (Figures S1 and S2). DEDTP consistently had positive posterior mean contrasts with EAA when other DAPs were fixed at 25th, 50th, and 75th quantiles with varying posterior precision (Figure S4). Little evidence of strong interactions among metabolites in this study was displayed in the resulting pairwise interaction curves and interaction contrasts (Figures S3 and S5). The MCMC trace plots accompanying the BKMR suite exhibited generally stable sampling behavior, with some early wandering apparent for GrimAge2 (Figure S6).

## 4. Discussion 

### 4.1. Principal Findings: Conventional Regression vs. BKMR Estimates for DAP and EAA Exposure–Response Functions

Upon completing the multivariable linear regression in this cross-sectional NHANES analysis, we found that there was limited evidence of linear associations between each DAP metabolite and EAA as all confidence intervals included the null. BKMR exhibited heterogeneous exposure–response patterns in individual, paired and joint mixtures instead of a general, uniform association with EAA. As mixture quantiles increased under HorvathAge and HannumAge, posterior mean overall mixture estimates also increased. Moderate increases were shown for PhenoAge, while overall mixture estimates either decreased or remained close to null for GrimAge and GrimAge2. However, the credible intervals accompanying these estimates generally included zero, which indicate consistent posterior uncertainty. In the primary models, DEDTP stood out among the DAPs as it had the highest PIP within each of the five clocks, although the magnitudes were low for GrimAge and GrimAge2 and were sensitive to MCMC chain length for GrimAge2.

The differences between the regression and BKMR results should not be interpreted as demonstrating that the linear models were incorrect or that BKMR establishes associations that the linear models failed to detect. Rather, the two approaches characterize different features of the exposure–outcome relationship, and the BKMR results should be considered exploratory evidence of potentially nonlinear patterns requiring replication. One further consideration when comparing estimates across outcomes is that the clocks themselves differ in how they were constructed. The first-generation clocks such as Horvath and Hannum were built using CpG sites to predict chronological age while second-generation clocks such as PhenoAge, GrimAge, and GrimAge2 were trained using established physiological stressors and clinical biomarkers relating to CpG sites that track cellular and tissue vulnerability [32]. Because these clocks incorporate different biological information, their associations with environmental exposures may differ, potentially contributing to the heterogeneity of estimates observed across clocks. Yin et al. similarly reported an organophosphate-health-outcome analysis in which linear regression models provided limited evidence of association, while BKMR characterized additional features of the exposure–response relationship [33]. The figures and tables from the BKMR analyses in our own study provide suggestions of nonlinear relationships between the organophosphate mixture and accelerated epigenetic aging. Upon removing the assumption of linearity, these outputs also exhibited potential nuances of importance regarding individual metabolites. This suggests that the exposure–outcome associations of urinary DAP metabolites not represented by single linear coefficients and EAA may be more fully characterized by adopting flexible mixture models than depending on conventional regressions.

A consistent and distinct feature observed in these clock analyses is that the strongest positive posterior mean mixture patterns were observed for first-generation clocks HorvathAge and HannumAge. The second-generation clocks PhenoAge, GrimAge, and GrimAge2 had less distinct positive posterior mean estimates or differing patterns. Differences in how each epigenetic clock was constructed may account in part for why estimates for individual and combined dialkylphosphates in the DAP panel diverged in direction across clocks. HorvathAge and HannumAge are first-generation clocks developed primarily to predict chronological age, whereas PhenoAge, GrimAge, and GrimAge2 are later-generation clocks that incorporate phenotypic, physiological, or mortality-related information [34]. These divergent patterns do not provide evidence for a uniform acceleration of epigenetic aging by DAP exposure. Instead, they suggest that any association between the DAP mixture and DNA methylation-derived aging measures may be clock-dependent. Because several credible intervals were wide and included the null, the observed curves should be interpreted as estimated exposure–response patterns rather than definitive evidence of either accelerated or decelerated biological aging. These patterns are illustrated in Figure 4, which displays the estimated overall mixture–response function for each epigenetic clock across increasing exposure quantiles.

Three previous analyses have examined organophosphate biomarkers in relation to DNA methylation-based aging in NHANES, and the present results are best understood against them. Cheng et al. [11] reported associations between urinary organophosphate metabolites and accelerated biological aging in two NHANES cohorts. Their discovery cohort of 9795 adults drew on the 2003–2008, 2011–2012, and 2015–2018 cycles and used an algorithm-based biological age computed from routine clinical chemistry rather than from DNA methylation. Their validation cohort of 2494 participants drew on the 1999–2002 cycles and used HorvathAge, HannumAge, PhenoAge, and GrimAge, so it overlaps in resource and in four of the five clocks with the present analysis. In that validation cohort, they reported positive associations for DMP with HannumAge and PhenoAge, for DEP with HannumAge, HorvathAge, and GrimAge, for DMTP with HorvathAge and GrimAge, and for DETP with all four clocks. The present analysis found no such associations, and the difference merits explanation rather than assertion. Four features of the two designs differ. First, that study evaluated four metabolites, DMP, DEP, DMTP, and DETP, whereas the present panel also includes DMDTP and DEDTP, and DEDTP carried the highest posterior inclusion probability in every clock-specific BKMR model here. Second, each metabolite there was modeled separately, whereas all six enter the present models simultaneously, so that every coefficient is mutually adjusted for the remaining five; with correlated exposures, a single-metabolite coefficient absorbs variance shared across the panel. Third, those associations were evaluated at a nominal threshold without correction across the metabolite–outcome grid, and most carried *p*-values between 0.01 and 0.05, whereas the present estimates were subjected to Benjamini–Hochberg correction across all 30 tests. Fourth, the exposure scale and covariate sets differ: concentrations there were log-transformed, whereas the present models use standardized concentrations and additionally adjust for urinary creatinine to account for variation in spot urine dilution. The analytic sample also differs substantially in size, since the present sample is restricted to the intersection of the DNA methylation and urinary pesticide subsamples among adults aged 50 years and older (*n* = 427), which yields correspondingly wider intervals. Khodasevich et al. [12] analyzed the NHANES 1999–2002 methylation resource directly, evaluating 64 environmental exposures, among them six urinary pesticide metabolites, against eight epigenetic clocks using survey-weighted models with Bonferroni correction. Significant associations in that study were confined to cadmium, lead, serum cotinine, and selected polychlorinated biphenyls and dioxins; no urinary pesticide metabolite reached significance. Song et al. [3] examined the same resource in 502 adults aged 50 years and older and reported suggestive indirect effects involving methylation-based measures at nominal significance that did not survive false-discovery-rate correction. A systematic review and meta-analysis of pesticide exposure and biological aging likewise reported no significant pooled association with Hannum clock acceleration, the only clock for which a pooled estimate was available across the four eligible studies (β = 0.27, 95% CI −0.25 to 0.79) [15]. Taken together, positive associations between dialkylphosphate metabolites and DNA methylation clocks in NHANES have been reported when metabolites are examined individually at nominal significance, whereas the analyses that have applied multiplicity correction, including the present one, have not found associations that survive it. The contribution of the present study is to show that the null result holds under a specification that differs on several axes at once: all six metabolites modeled jointly with mutual adjustment, chronological age and smoking status included as covariates, false-discovery-rate correction applied across the full test grid, and flexible mixture modeling permitted to detect nonlinearity and pairwise interaction that single-pollutant linear models cannot represent. Whether the divergence reflects the mutual adjustment, the multiplicity correction, the reduced sample available at the intersection of the two subsamples, or some combination of these cannot be resolved with these data, and replication in cohorts with both repeated exposure measurements and methylation data would be required to settle it.

### 4.2. Mixture Component Importance and Clock Variation

In the primary models, DEDTP had the highest PIP within each clock, as displayed in Figure 5 and Table 3: HannumAge (0.868), HorvathAge (0.776), PhenoAge (0.616), GrimAge (0.440), and GrimAge2 (0.214). This ordering was reproduced at 20,000 iterations for the first four clocks but not for GrimAge2, where DEDTP and DMDTP were effectively indistinguishable (Table S2). While DEDTP stood out among the six metabolites, the PIP values on their own do not establish associations with EAA. The single-DAP summaries in Figure S4 are consistent with this ordering: the posterior mean contrast for DEDTP was positive in all five clocks when the remaining five metabolites were held at their 25th, 50th, and 75th percentiles, and the corresponding credible intervals were narrower for HorvathAge and HannumAge than for the later-generation clocks. Because numerical posterior summaries for these contrasts are not tabulated, no conclusion is drawn here from whether an individual credible interval excludes the null. The remaining DAP metabolites showed an inconsistent direction and broad posterior uncertainty. The contrasting directions of the estimated exposure–response relationships shown in Figure 4 suggest that outcomes are clock-specific, although they do not by themselves establish the biological mechanisms underlying those differences. Furthermore, the univariate and bivariate results shown in Figures S1 and S2 indicate suggestions of nonlinearity and heterogeneity instead of a general linear outcome, but uncertainty increases as the exposure level increases. This is most notably seen in HorvathAge and HannumAge for DEDTP. However, because the posterior distributions for several of these contrasts retained appreciable mass on both sides of the null, the direction of the effect is estimated with limited posterior certainty rather than being ruled out. Therefore, the BKMR analyses suggest possible nonlinear and clock-dependent exposure–response patterns, but the posterior uncertainty does not support definitive conclusions regarding either the presence or direction of associations for many contrasts. These findings should be considered hypothesis-generating and require replication in larger independent populations. The evidence of strong interaction among DAP metabolites in Figure S5 is limited and coincides with the weak to moderate correlations produced in the Spearman correlation, while a lack of problematic multicollinearity was suggested by the VIF values in Table S1. The limited evidence for pairwise interaction suggests that strong interactions among individual DAP metabolites were not clearly supported in this sample; however, this should not be interpreted as evidence that the observed patterns are necessarily attributable to a specific form of combined exposure. This could be confirmed further by analyzing populations with higher exposure levels.

### 4.3. Strengths and Limitations

A strength of this study is that the data were drawn from NHANES, whose established complex survey design supports design-based estimation for the sampled subpopulation. Survey weighting was incorporated into the descriptive and conventional regression analyses to account for the NHANES sampling design; however, it is noteworthy that survey weighting decreases power relative to an otherwise comparable unweighted analysis. Because the BKMR models were unweighted, population-level generalizability should not be extended to those analyses. As a methodological strength, the inclusion of BKMR analyses allowed for the assessment of a mixture of organophosphates as well as possible nonlinear relationships and interactions rather than focusing on individual DAPs at a time. The use of five epigenetic clocks permitted the evaluation of whether estimated DAP associations differed across clock-specific EAA measures. This multi-clock approach provided a broader assessment of heterogeneity across DNA methylation-derived aging measures without assuming that the clocks represent interchangeable measures of a single biological aging process. Spearman correlations among paired DAP metabolites were mostly weak to moderate.

Posterior precision varied across outcomes, metabolites, and exposure levels. Several individual metabolite contrasts were estimated relatively precisely, while uncertainty generally increased at higher exposure levels. EAA is an algorithm-derived, clock-specific molecular phenotype and should not be equated directly with the rate of organismal biological aging. Due to NHANES data being cross-sectional, a limitation of this study is that neither causality nor temporal ordering can be established. The final analytic sample (*n* = 427) after covariate adjustment limited posterior precision, especially at the upper end of the exposure distribution. Another limitation concerns the composition of the analytic sample. The NHANES DNA methylation ancillary study selected all eligible non-Hispanic Black, Mexican American, other Hispanic, and other-race participants aged 50 years and older but only approximately half of eligible non-Hispanic White participants, so racial and ethnic minority groups are over-represented in the unweighted analytic sample (non-Hispanic White 47.3%, Mexican American 24.4%, non-Hispanic Black 18.3%, other Hispanic 5.4%, and other race 4.7%). Survey weighting recovers nationally representative proportions for the descriptive and conventional regression analyses, as shown in Table 1, but the BKMR models were unweighted and therefore reflect the composition of the subsample rather than that of the United States population. The BKMR results should accordingly be interpreted as associations within the analytic sample rather than as nationally representative estimates. Furthermore, the interpretation of metabolite-specific BKMR results concerning DEDTP should consider differences in analytical detection frequency as DEDTP had the lowest detection frequency (27.4%), ultimately resulting in a reduced DEDTP exposure resolution. Nonspecific metabolites such as DAPs limit the attribution of association outcomes to specific pesticides since they are from an array of parent organophosphate pesticides. Lastly, unaccounted lifestyle and environmental confounding factors aside from the covariates included in this study may cause confounding issues and should not be excluded until proven to be negligible. Although smoking status was adjusted for, smoking intensity measures such as pack-years and serum cotinine were not, and alcohol consumption, diet, physical activity, and occupational exposure were likewise unmodeled. Leukocyte cell-type proportions were not adjusted for in the primary models, although adding them in sensitivity analysis did not change the substantive conclusion (Table S4); because the BKMR models were not refitted with cell-type adjustment, residual confounding of the mixture results by leukocyte composition cannot be excluded. Future directions include larger longitudinal cohorts, parent pesticide biomarkers in addition to DAP metabolites, and mechanistic studies directly observing DNA methylation patterns and pathways upon exposure.

While DAPs have shared exposure sources comprising the diet, agriculture, and occupational/residential pesticide usage, these urinary metabolites such as DMP are non-specific. Dialkylphosphates are the results of metabolic breakdowns from parent organophosphate pesticides such as malathion or preformed DAPs within the environment [35]. Also, the metabolites in the DAP panel have a short half-life, ranging from 24 to 72 h, so a single specimen reflects recent contact rather than usual exposure and is subject to substantial within-person variability. Because of within-person temporal variability from these single spot urine specimens, subsequent exposure misclassification could contribute to the attenuation of exposure–outcome estimates. Because ingestion is a main route of exposure and dietary intake varies from day to day, a spot specimen captures only a narrow window of that variation, which further increases exposure measurement error [35].

## 5. Conclusions

In this cross-sectional analysis of older U.S. adults, conventional regression provided limited evidence of associations between urinary DAP metabolites and epigenetic age acceleration, whereas exploratory BKMR analyses suggested potentially nonlinear and clock-dependent exposure–response patterns. In the primary models, DEDTP had the highest posterior inclusion probability within each clock, although its magnitude was low for the later-generation clocks and was not reproduced for GrimAge2 in the longer sensitivity run. The direction of the estimated mixture associations differed across epigenetic clocks, and substantial posterior uncertainty was present for many estimates. Accordingly, these findings do not establish that the urinary DAP mixture, or interactions among its components, accelerates biological aging. Rather, they suggest that DAP metabolite concentrations may be associated with selected DNA methylation-derived age measures in ways that differ according to clock construction. These results should be considered hypothesis-generating and warrant evaluation in larger longitudinal studies incorporating repeated exposure measurements and temporally resolved DNA methylation assessments. Furthermore, these findings illustrate the value of flexible mixture models for characterizing environmental exposure–response patterns while providing a foundation for future studies with larger analytic samples.

## Figures and Tables

**Figure 1:**
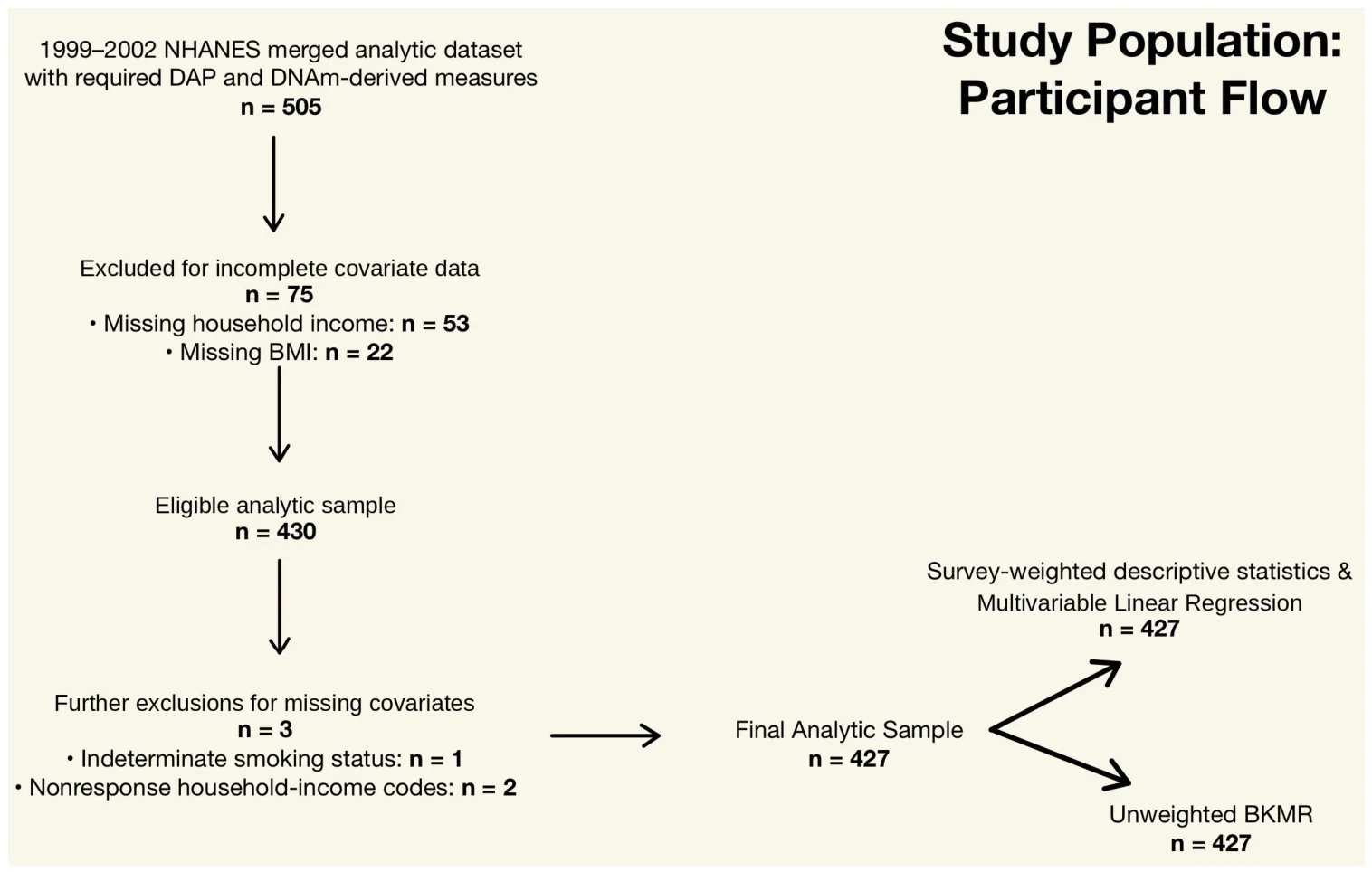
Participant flow for study population. *n* = number of participants; DAP = dialkylphosphate; DNAm = DNA methylation; BKMR = Bayesian Kernel Machine Regression.

**Figure 2:**
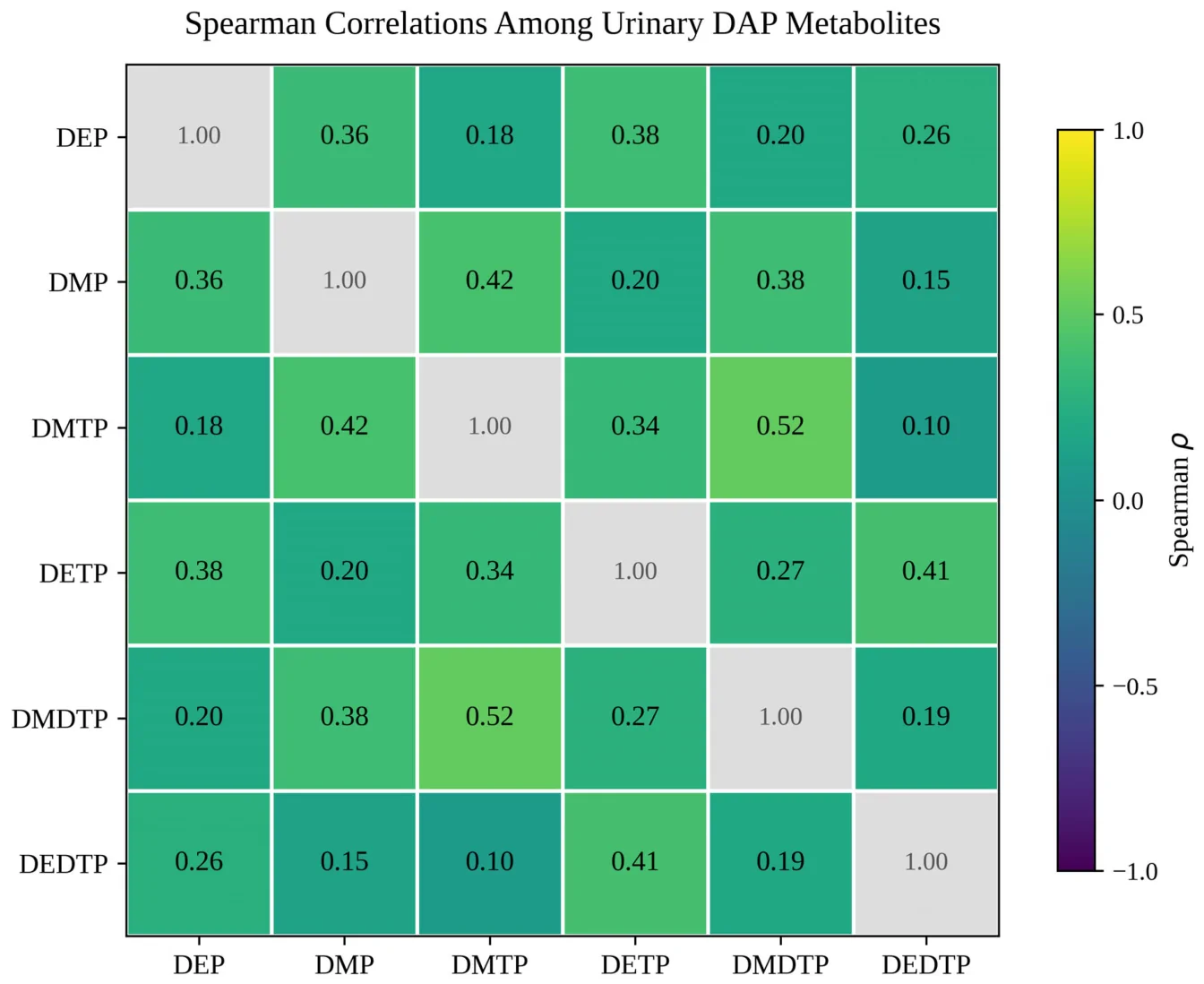
Spearman correlation among DEP, DMP, DMTP, DETP, DMDTP, and DEDTP metabolites. (DEP—diethylphosphate, DMP—dimethylphosphate, DMTP—dimethylthiophosphate, DETP—diethylthiophosphate, DMDTP—dimethyldithiophosphate, DEDTP—diethyldithiophosphate). The color scale spans the full range of the Spearman coefficient, from −1 to 1. The diagonal is shaded gray because it is fixed at 1.00 by construction. Correlations were classified by absolute magnitude as weak (|ρ| < 0.4), moderate (0.4 ≤ |ρ| < 0.8), or strong (|ρ| ≥ 0.8). All observed off-diagonal coefficients fell between 0.10 and 0.52.

**Figure 4:**
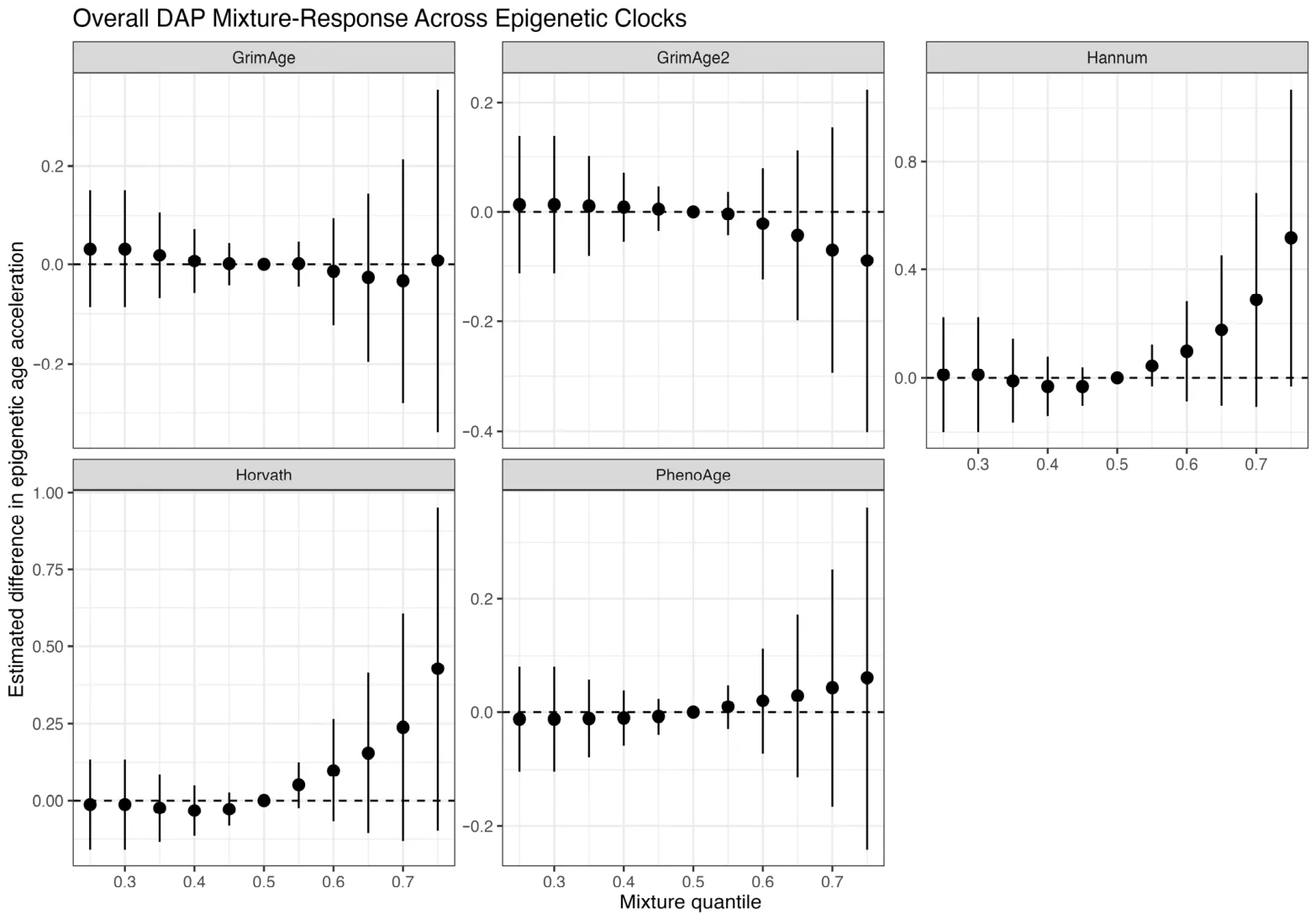
Overall mixture–response association between the DAP panel and epigenetic age acceleration. Points are posterior mean estimated differences in epigenetic age acceleration comparing all six DAP metabolites jointly at the indicated mixture quantile with all six metabolites held at their 50th percentile; vertical bars are 95% credible intervals. Estimates are from the unweighted primary 5000-iteration BKMR models. Panels are arranged alphabetically (top row: GrimAge, GrimAge2, Hannum; bottom row: Horvath, PhenoAge); the panel labels Hannum and Horvath denote HannumAge and HorvathAge. Vertical axis scales are panel-specific, so magnitudes should not be compared directly across panels. The horizontal dashed line marks zero, i.e., no difference from the reference in which all six metabolites are held at their 50th percentile.

**Figure 5:**
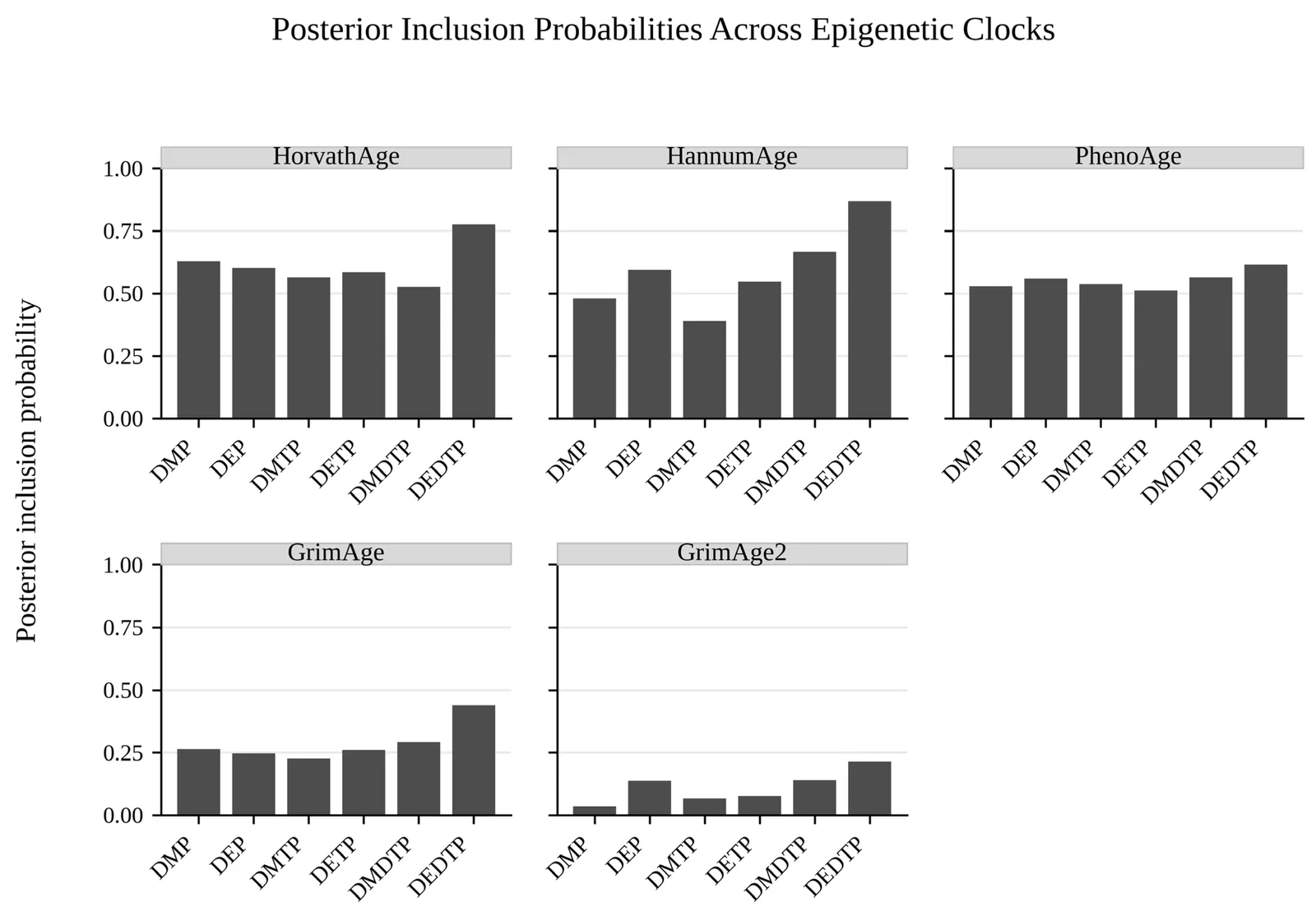
BKMR posterior inclusion probabilities for DAP panel vs. accelerated epigenetic aging, from the primary 5000-iteration models. Panels are ordered from first-generation to later-generation clocks. PIP values range from 0 to 1, with higher values indicating greater posterior support for inclusion of a metabolite in the fitted BKMR model; values are compared across metabolites within each clock-specific panel. Numeric values are given in Table 3. DEP—diethylphosphate, DMP—dimethylphosphate, DMTP—dimethylthiophosphate, DETP—diethylthiophosphate, DMDTP—dimethyldithiophosphate, DEDTP—diethyldithiophosphate.

**Table 1:** Baseline study sample characteristics of subpopulation.

**Continuous Variables**	
Sample Characteristics	Mean (SE)
Age (years)	60.78 (0.67)
BMI (kg/m^2^)	28.79 (0.45)
Creatinine (mg/dL)	106.73 (4.44)
**Unadjusted Exposure Variables (µg/L)**	
DMP	2.84 (0.31)
DEP	2.75 (0.40)
DMTP	9.50 (1.34)
DETP	1.10 (0.14)
DMDTP	2.74 (0.63)
DEDTP	0.21 (0.03)
**Creatinine-adjusted Exposure Variables (µg/g)**	
DMP	3.22 (0.29)
DEP	2.51 (0.28)
DMTP	9.47 (1.22)
DETP	1.32 (0.18)
DMDTP	2.92 (0.52)
DEDTP	0.36 (0.09)
**Categorical Variables**	
**Sex**	
Male	47.02%
Female	52.98%
**Ethnicity**	
Non-Hispanic White	80.61%
Non-Hispanic Black	6.87%
Mexican American	3.27%
Other Race—Including Multi-Racial	4.92%
Other Hispanic	4.33%
**Education Level**	
Less Than 9th Grade	6.79%
9–12th Grade (No Diploma)	15.80%
High School Graduate/GED or Equivalent	24.37%
Some College or AA degree	26.32%
College Graduate or Above	26.73%
**Income (USD)**	
$0 to $4999	1.19%
$5000 to $9999	4.59%
$10,000 to $14,999	5.39%
$15,000 to $19,999	6.82%
$20,000 to $24,999	8.15%
$25,000 to $34,999	11.63%
$35,000 to $44,999	9.25%
$45,000 to $54,999	10.25%
$55,000 to $64,999	10.23%
$65,000 to $74,999	7.29%
$75,000 and over	25.13%
Over $20,000 (amount not specified)	0.08%
**Smoking**	
Never	40.88%
Former	40.77%
Current	18.35%

BMI = Body Mass Index, SE = Standard Error, AA = Associate of Arts, DMP—dimethylphosphate, DEP—diethylphosphate, DMTP—dimethylthiophosphate, DETP—diethylthiophosphate, DMDTP—dimethyldithiophosphate, DEDTP—diethyldithiophosphate.

**Table 3:** BKMR posterior inclusion probabilities for DAP panel vs. accelerated epigenetic aging.

Variable	HorvathAge	HannumAge	PhenoAge	GrimAge	GrimAge2
DMP	0.6288	0.4804	0.5288	0.2636	0.0344
DEP	0.6024	0.5952	0.5584	0.2464	0.1368
DMTP	0.5632	0.3904	0.5380	0.2260	0.0668
DETP	0.5852	0.5476	0.5124	0.2604	0.0768
DMDTP	0.5272	0.6660	0.5640	0.2928	0.1392
DEDTP	0.7756	0.8680	0.6156	0.4396	0.2144

DAP—dialkylphosphate, DEP—diethylphosphate, DMP—dimethylphosphate, DMTP—dimethylthiophosphate, DETP—diethylthiophosphate, DMDTP—dimethyldithiophosphate, DEDTP—diethyldithiophosphate.

## Data Availability

The original data presented in this study are openly available in the National Health and Nutrition Examination Survey (NHANES) repository at https://wwwn.cdc.gov/nchs/nhanes/ (accessed on 12 March 2026). The urinary pesticide metabolite files are LAB26PP (1999–2000) and L26PP_B (2001–2002), and the DNA methylation and epigenetic biomarker files are DNMEPI (1999–2000) and DNMEPI_B (2001–2002). See also https://wwwn.cdc.gov/nchs/nhanes/continuousnhanes/overview.aspx?BeginYear=2001 (accessed on 12 March 2026).

## Supplementary Materials

The following supporting information can be downloaded at https://www.mdpi.com/article/10.3390/jox16060182/s1, Methods S1, BKMR implementation and MCMC analysis; Figure S1, Univariate DAP exposure–response functions across epigenetic clocks; Figure S2, Bivariate DAP exposure–response functions across epigenetic clocks; Figure S3, Pairwise DAP interaction functions across epigenetic clocks; Figure S4, Single-DAP effect summaries across epigenetic clocks; Figure S5, DAP interaction summaries across epigenetic clocks; Figure S6, MCMC trace plots for primary BKMR models across epigenetic clocks; Figure S7, MCMC trace plots for sensitivity BKMR models across epigenetic clocks; Table S1, Variance inflation factors for urinary DAP metabolites from multivariable regression models; Table S2, Comparison between primary and sensitivity analysis BKMR results; Table S3, Analytical limits of detection and detection frequencies for urinary DAP metabolites in the final analytical sample; Table S4, Sensitivity analyses adjusting for leukocyte cell-type proportions and using collapsed household income; Table S5, Unweighted and survey-weighted descriptive statistics for the final analytic sample.
