# Supplementary material for: Associations of Organophosphate Pesticide Metabolite Mixtures with Epigenetic Age Acceleration
Source: J Xenobiot. 2026 Sep 25;16(6):182. doi: 10.3390/jox16060182 (PMC13706362; doi:10.3390/jox16060182)
Supplement: Supplementary file 1 [file jox-16-00182-s001.zip › jox-4515329-supplementary.pdf]

Supplementary Materials: Associations of Organophosphate Pesticide Metabolite Mixtures with Epigenetic Age Acceleration

Taújay Davis and Emmanuel Obeng-Gyasi

Supplementary Methods S1. BKMR implementation and MCMC analysis.

The R version 4.5.1 using R Studio 2025.09.2+418 (Posit Software, PBC) was used for all BKMR analyses in this study. The bkmr package (version 0.2.2) was used to fit the models with the bkmr::kmbayes() function, and the fields package (version 17.3) was used to generate predictive-process knots. The six DAP concentrations were converted to z-scores with scale() immediately prior to modeling; no logarithmic or other transformation was applied before standardization, so all exposure coefficients and contrasts are expressed per one standard deviation of the concentration scale. The covariates were chronological age, sex, ethnicity, education, income, BMI, urinary creatinine, and smoking status. Covariates entered the model matrix in this order, so the coefficient denoted $\beta 1$ in Figures S6 and S7 is the coefficient for chronological age. Component-wise variable selection utilized varsel = TRUE for implementation. A single MCMC chain was run for each clock-specific model, so convergence was assessed by visual inspection of trace plots and by comparing posterior inclusion probabilities and overall mixture-response summaries across chain lengths and random seeds rather than by multi-chain diagnostics such as the Gelman–Rubin statistic. There were 50 predictive-process knots using fields::cover.design() with a random seed of 10 for the knot production. There were 5,000 primary MCMC iterations, with the first 2,500 discarded as burn-in and iterations 2,501 to 5,000 retained for all posterior summaries and PIP analysis. Primary clock-specific random seeds consisted of 101, 102, 103, 104, and 105 for HorvathAge, HannumAge, PhenoAge, GrimAge, and GrimAge2 models, respectively. An additional independent GrimAge2 model was fitted with a random seed of 905 and 20,000 iterations to assess whether the reordering of components observed for that clock was seed-dependent; this run likewise produced uniformly low posterior inclusion probabilities for all six metabolites and an overall mixture-response function close to the null. There were 20,000 iterations performed for the sensitivity analyses, and its posterior sample utilized the iterations ranging from 10,001 to 20,000. There was no thinning for inference methods in this study. Trace plots were thinned for display only: every 5th draw after iteration 1,000 for the primary models (800 plotted draws) and every 10th draw from iterations 10,001 to 20,000 for the sensitivity analyses (1,000 plotted draws). Thinning was applied to the plots alone and not to the posterior summaries. Default bkmr prior specifications were used: a.p0 = 1 and b.p0 = 1 for the variable-inclusion probability prior, r.prior = ″invunif″, a.sigsq = b.sigsq = 0.001. The stability of the MCMC iterations was evaluated by visual inspection of trace plots and comparisons of posterior inclusion probabilities and overall mixture-response summaries between the primary and sensitivity runs.

Table S1. Variance inflation factors for urinary DAP metabolites from multivariable regression models.

Exposure Variable	Variance Inflation Factor
DMP	1.45
DEP	1.20
DMTP	1.56
DETP	1.21
DMDTP	1.37
DEDTP	1.11

Values range from 1.11 to 1.56. DEP, DMP, DMTP, DETP, DMDTP, and DEDTP metabolites (DEP –diethylphosphate, DMP—dimethylphosphate, DMTP—dimethylthiophosphate, DETP –diethylthiophosphate, DMDTP—dimethyldithiophosphate, DEDTP—diethyldithiophosphate).

Table S2. Comparison between primary and sensitivity analysis BKMR results.

Epigenetic Clock	DAP	PIP (5k iterations)	PIP (20k iterations)	Absolute Difference
HorvathAge	DMP	0.629	0.478	0.151
HorvathAge	DEP	0.602	0.514	0.088
HorvathAge	DMTP	0.563	0.393	0.171
HorvathAge	DETP	0.585	0.500	0.085
HorvathAge	DMDTP	0.527	0.366	0.161
HorvathAge	DEDTP	0.776	0.691	0.084
HannumAge	DMP	0.480	0.488	0.008
HannumAge	DEP	0.595	0.598	0.003
HannumAge	DMTP	0.390	0.424	0.034
HannumAge	DETP	0.548	0.571	0.023
HannumAge	DMDTP	0.666	0.665	0.001
HannumAge	DEDTP	0.868	0.827	0.041
PhenoAge	DMP	0.529	0.340	0.189
PhenoAge	DEP	0.558	0.456	0.102
PhenoAge	DMTP	0.538	0.356	0.182
PhenoAge	DETP	0.512	0.289	0.223
PhenoAge	DMDTP	0.564	0.415	0.149
PhenoAge	DEDTP	0.616	0.488	0.128
GrimAge	DMP	0.264	0.070	0.194
GrimAge	DEP	0.246	0.101	0.146
GrimAge	DMTP	0.226	0.115	0.111
GrimAge	DETP	0.260	0.118	0.142
GrimAge	DMDTP	0.293	0.137	0.155
GrimAge	DEDTP	0.440	0.392	0.047
GrimAge2	DMP	0.034	0.144	0.109
GrimAge2	DEP	0.137	0.146	0.009
GrimAge2	DMTP	0.067	0.197	0.131
GrimAge2	DETP	0.077	0.168	0.092
GrimAge2	DMDTP	0.139	0.251	0.112
GrimAge2	DEDTP	0.214	0.246	0.032

PIP values range from 0 to 1, with higher values indicating greater posterior support for inclusion of a metabolite in the fitted BKMR model. Absolute differences were computed from the unrounded posterior inclusion probabilities and may therefore differ by 0.001 from the difference of the rounded values displayed. DEP—diethylphosphate, DMP—dimethylphosphate, DMTP—dimethylthiophosphate, DETP—diethylthiophosphate, DMDTP—dimethyldithiophosphate, DEDTP—diethyldithiophosphate.

Table S3. Analytical limits of detection and detection frequencies for urinary DAP metabolites in the final analytical sample.

DAP Metabolite	1999-2000 LOD (µg/L)	2001-2002 LOD (µg/L)	N	Below LOD, n (%)	Detected, n (%)
DMP	0.58	0.50	427	213 (49.9)	214 (50.1)
DEP	0.20	0.20	427	188 (44.0)	239 (56.0)
DMTP	0.18	0.40	427	187 (43.8)	240 (56.2)

DETP	0.09	0.10	427	131 (30.7)	296 (69.3)
DMDTP	0.08	0.10	427	246 (57.6)	181 (42.4)
DEDTP	0.05	0.10	427	310 (72.6)	117 (27.4)

N = final analytical sample, LOD = Limit of Detection, n = number of participants. DEP, DMP, DMTP, DETP, DMDTP, and DEDTP metabolites (DEP –diethylphosphate, DMP—dimethylphosphate, DMTP—dimethylthiophosphate, DETP –diethylthiophosphate, DMDTP—dimethyldithiophosphate, DEDTP—diethyldithiophosphate).

Table S4. Sensitivity analyses adjusting for leukocyte cell-type proportions and using collapsed household income.

Epigenetic Clock	DAP	Cell-Type-Adjusted β (95% CI)	p	q	Collapsed-Income β (95% CI)	p	q
HorvathAge	DMP	0.28 (-0.42 to 0.98)	0.427	0.809	0.47 (-0.40 to 1.34)	0.275	0.751
HorvathAge	DEP	-0.18 (-0.68 to 0.31)	0.455	0.809	-0.29 (-0.88 to 0.29)	0.314	0.785
HorvathAge	DMTP	0.02 (-0.81 to 0.85)	0.961	0.989	0.06 (-0.78 to 0.90)	0.887	0.918
HorvathAge	DETP	0.25 (-0.67 to 1.16)	0.588	0.841	0.19 (-0.71 to 1.10)	0.665	0.894
HorvathAge	DMDTP	0.13 (-0.20 to 0.47)	0.415	0.809	0.11 (-0.21 to 0.43)	0.482	0.894
HorvathAge	DEDTP	0.31 (-0.54 to 1.16)	0.466	0.809	0.37 (-0.47 to 1.22)	0.375	0.805
HannumAge	DMP	0.22 (-0.31 to 0.74)	0.405	0.809	0.44 (-0.22 to 1.10)	0.181	0.751
HannumAge	DEP	-0.18 (-0.67 to 0.31)	0.451	0.809	-0.54 (-1.07 to -0.01)	0.046	0.676
HannumAge	DMTP	0.04 (-0.95 to 1.03)	0.931	0.989	0.17 (-0.98 to 1.33)	0.759	0.894
HannumAge	DETP	0.05 (-0.75 to 0.86)	0.890	0.989	0.04 (-0.89 to 0.97)	0.928	0.928
HannumAge	DMDTP	0.23 (-0.48 to 0.94)	0.512	0.809	0.23 (-0.59 to 1.05)	0.572	0.894
HannumAge	DEDTP	0.00 (-0.61 to 0.61)	0.989	0.989	0.06 (-0.55 to 0.67)	0.834	0.894
PhenoAge	DMP	0.29 (-0.48 to 1.07)	0.447	0.809	0.47 (-0.38 to 1.31)	0.267	0.751
PhenoAge	DEP	-0.28 (-1.01 to 0.45)	0.437	0.809	-0.64 (-1.21 to -0.07)	0.029	0.676
PhenoAge	DMTP	-0.81 (-2.15 to 0.52)	0.222	0.809	-0.62 (-2.03 to 0.79)	0.376	0.805
PhenoAge	DETP	0.55 (-0.46 to 1.57)	0.273	0.809	0.22 (-0.87 to 1.31)	0.680	0.894
PhenoAge	DMDTP	-0.37 (-0.85 to 0.10)	0.121	0.728	-0.23 (-0.93 to 0.47)	0.510	0.894
PhenoAge	DEDTP	-0.22 (-0.83 to 0.39)	0.472	0.809	-0.16 (-0.78 to 0.45)	0.596	0.894
GrimAge	DMP	0.09 (-0.51 to 0.68)	0.772	0.989	0.10 (-0.59 to 0.80)	0.763	0.894
GrimAge	DEP	0.37 (0.10 to 0.65)	0.009	0.264	-0.06 (-0.41 to 0.29)	0.742	0.894
GrimAge	DMTP	-0.46 (-0.96 to 0.05)	0.077	0.576	-0.45 (-1.00 to 0.11)	0.112	0.676
GrimAge	DETP	-0.49 (-1.25 to 0.27)	0.200	0.809	-0.57 (-1.49 to 0.34)	0.207	0.751
GrimAge	DMDTP	-0.00 (-0.30 to 0.29)	0.974	0.989	0.17 (-0.14 to 0.48)	0.266	0.751
GrimAge	DEDTP	-0.10 (-0.51 to 0.32)	0.640	0.872	-0.05 (-0.46 to 0.36)	0.802	0.894
GrimAge2	DMP	0.08 (-0.56 to 0.72)	0.797	0.989	0.09 (-0.74 to 0.91)	0.828	0.894
GrimAge2	DEP	0.24 (-0.02 to 0.49)	0.072	0.576	-0.25 (-0.56 to 0.06)	0.106	0.676
GrimAge2	DMTP	-0.60 (-1.19 to -0.01)	0.045	0.576	-0.55 (-1.24 to 0.14)	0.113	0.676
GrimAge2	DETP	-0.21 (-0.91 to 0.49)	0.547	0.821	-0.30 (-1.26 to 0.67)	0.532	0.894
GrimAge2	DMDTP	0.03 (-0.30 to 0.36)	0.862	0.989	0.23 (-0.13 to 0.59)	0.202	0.751
GrimAge2	DEDTP	-0.14 (-0.55 to 0.27)	0.486	0.809	-0.10 (-0.50 to 0.30)	0.600	0.894

Primary-model estimates for the same 30 metabolite-clock combinations are given in Table 2 of the main text. All models are survey-weighted (NHANES strata, primary sampling units, and survey weights) and enter all six standardized DAP metabolites simultaneously, so each β is mutually adjusted for the other five metabolites and is expressed per one-standard-deviation increase in the corresponding urinary concentration. The cell-type-adjusted model is the primary model plus the NCHS-derived proportions of CD8+ T cells, CD4+ T cells, natural killer cells, B cells, and monocytes, with neutrophils omitted as the referent composition. The collapsed-income model

replaces the 12-category income variable with a binary indicator of below versus at or above $20,000, which also resolves the NHANES code, denoting only that household income exceeded $20,000. q = Benjamini–Hochberg false-discovery-rate q-value computed within each model across its own 30 tests. No association was significant after correction in either sensitivity model (smallest q = 0.264 and 0.676, respectively). Design degrees of freedom = 29. DEP—diethylphosphate, DMP—dimethylphosphate, DMTP—dimethylthiophosphate, DETP—diethylthiophosphate, DMDTP—dimethyldithiophosphate, DEDTP—diethyldithiophosphate.

Table S5. Unweighted and survey-weighted descriptive statistics for the final analytic sample.

Characteristic	Unweighted Mean (SD) or n (%)	Survey-Weighted Mean (SE) or %
**Continuous variables**		
Age (years)	62.92 (10.41)	60.78 (0.67)
BMI (kg/m²)	28.80 (5.92)	28.79 (0.45)
Urinary creatinine (mg/dL)	107.97 (65.41)	106.73 (4.44)
**Unadjusted urinary DAP concentrations (μg/L)**		
DMP	3.22 (5.89)	2.84 (0.31)
DEP	3.02 (7.64)	2.75 (0.40)
DMTP	9.27 (23.85)	9.50 (1.34)
DETP	1.34 (3.21)	1.10 (0.14)
DMDTP	2.55 (7.66)	2.74 (0.63)
DEDTP	0.22 (0.53)	0.21 (0.03)
**Creatinine-adjusted urinary DAP concentrations (μg/g)**		
DMP	3.45 (5.49)	3.22 (0.29)
DEP	2.85 (6.66)	2.51 (0.28)
DMTP	9.71 (29.97)	9.47 (1.22)
DETP	1.53 (3.21)	1.32 (0.18)
DMDTP	3.01 (8.03)	2.92 (0.52)
DEDTP	0.33 (1.02)	0.36 (0.09)
**Sex**		
Male	215 (50.35%)	47.02%
Female	212 (49.65%)	52.98%
**Ethnicity**		
Non-Hispanic White	202 (47.31%)	80.61%
Non-Hispanic Black	78 (18.27%)	6.87%
Mexican American	104 (24.36%)	3.27%
Other Race—Including Multi-Racial	20 (4.68%)	4.92%
Other Hispanic	23 (5.39%)	4.33%
**Education Level**		
Less Than 9th Grade	82 (19.20%)	6.79%
9–12th Grade (No diploma)	82 (19.20%)	15.80%
High School Grade/GED or Equivalent	91 (21.31%)	24.37%
Some College or AA degree	91 (21.31%)	26.32%
College Graduate or Above	81 (18.97%)	26.73%
**Income (USD)**		
$0 to $4,999	8 (1.87%)	1.19%

$5,000 to $9,999	22 (5.15%)	4.59%
$10,000 to $14,999	34 (7.96%)	5.39%
$15,000 to $19,999	41 (9.60%)	6.82%
$20,000 to $24,999	45 (10.54%)	8.15%
$25,000 to $34,999	64 (14.99%)	11.63%
$35,000 to $44,999	39 (9.13%)	9.25%
$45,000 to $54,999	42 (9.84%)	10.25%
$55,000 to $64,999	35 (8.20%)	10.23%
$65,000 to $74,999	22 (5.15%)	7.29%
$75,000 and Over	73 (17.10%)	25.13%
Over $20,000 (amount not specified)	2 (0.47%)	0.08%
**Smoking**		
Never	190 (44.50%)	40.88%
Former	168 (39.34%)	40.77%
Current	69 (16.16%)	18.35%

n = 427 for all variables. Unweighted statistics are arithmetic means with standard deviations for continuous variables and counts with column percentages for categorical variables. Survey-weighted statistics use the NHANES strata, primary sampling units, and the WTDN4YR four-year DNA methylation subsample weight, and are reported as means with design-based standard errors or as weighted percentages; these are the values shown in Table 1 of the main text. The two columns differ most for race/ethnicity because the NHANES DNA methylation ancillary study sampled all eligible non-Hispanic Black, Mexican American, other Hispanic, and other-race participants aged 50 years and older but only approximately half of eligible non-Hispanic White participants. Unweighted statistics describe the sample represented in the unweighted Bayesian kernel machine regression models; survey-weighted statistics describe the population targeted by the conventional regression models. Percentages may not sum to 100 because of rounding. SD = standard deviation, SE = standard error, BMI = body mass index, AA = Associate of Arts, DMP—dimethylphosphate, DEP—diethylphosphate, DMTP—dimethylthiophosphate, DETP—diethylthiophosphate, DMDTP—dimethyldithiophosphate, DEDTP—diethyldithiophosphate.

Figure S1A. Univariate DAP Exposure–Response: HorvathAge

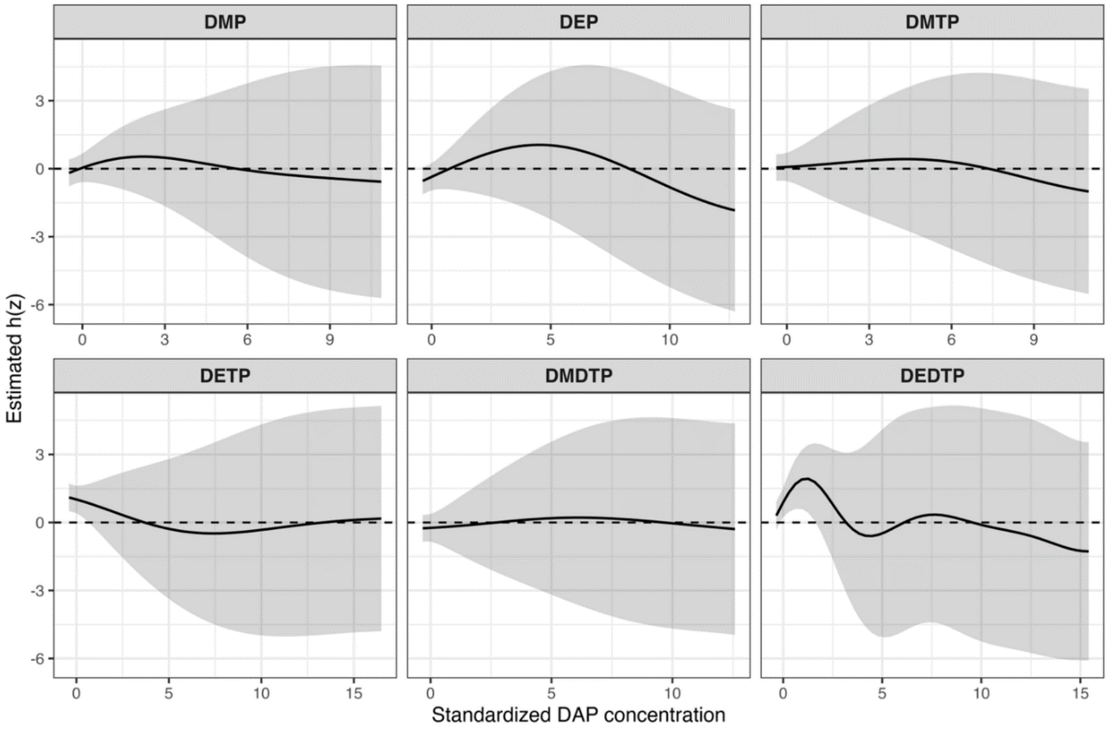

Figure S1B. Univariate DAP Exposure–Response: HannumAge

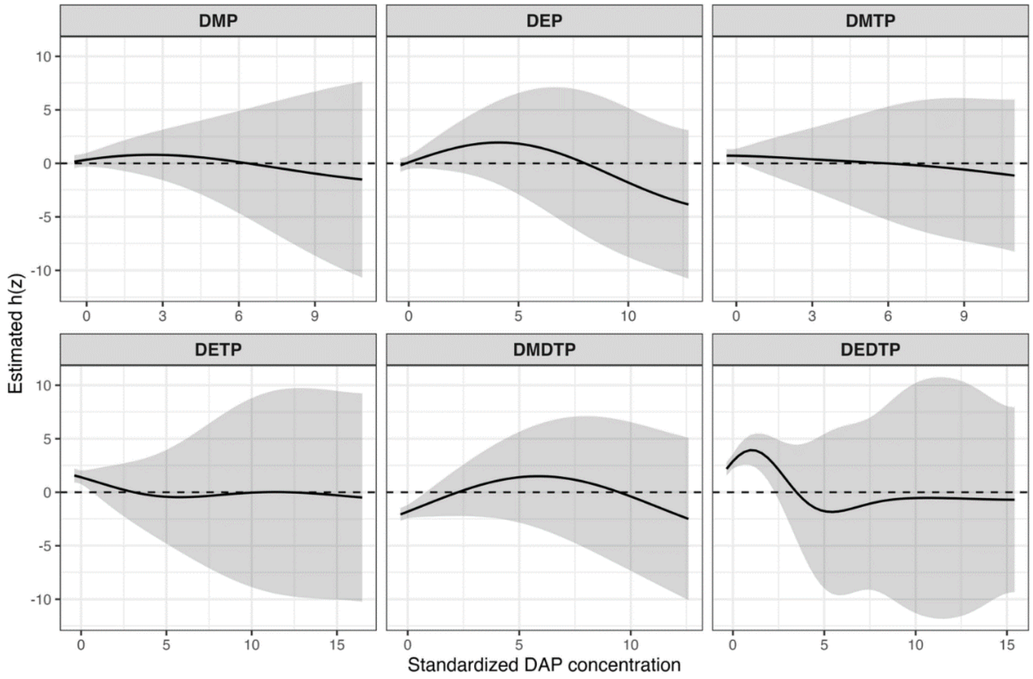

Figure S1C. Univariate DAP Exposure–Response: PhenoAge

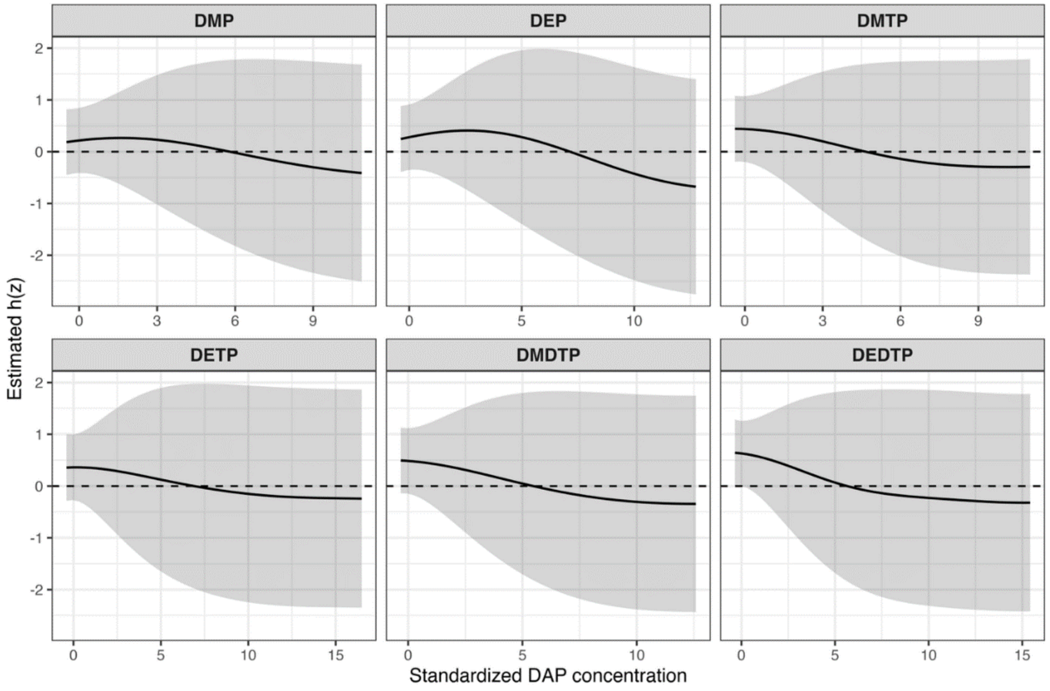

Figure S1D. Univariate DAP Exposure–Response: GrimAge

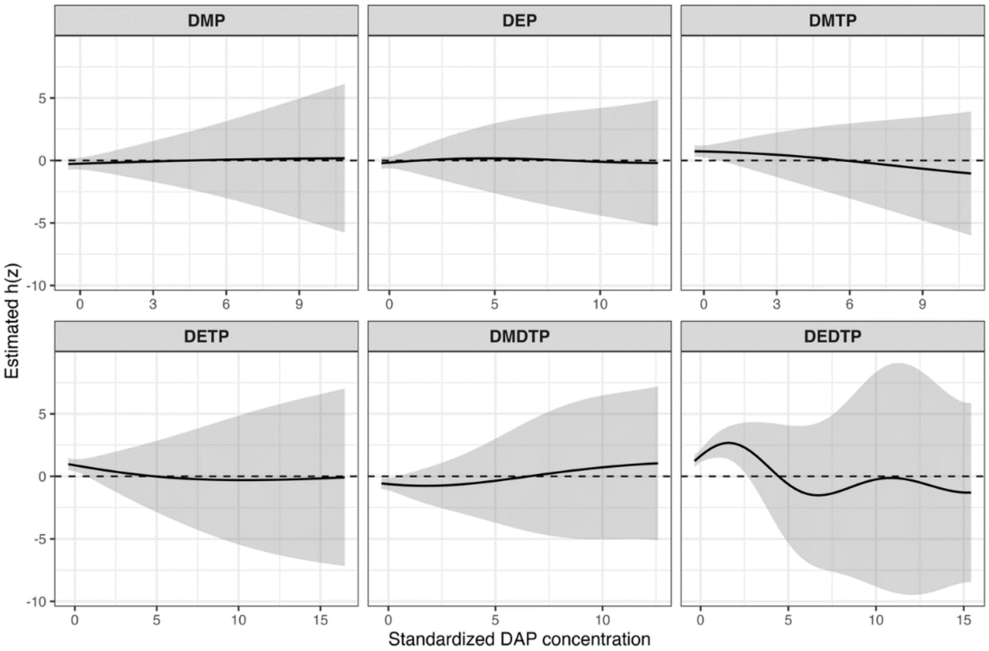

Figure S1E. Univariate DAP Exposure–Response: GrimAge2

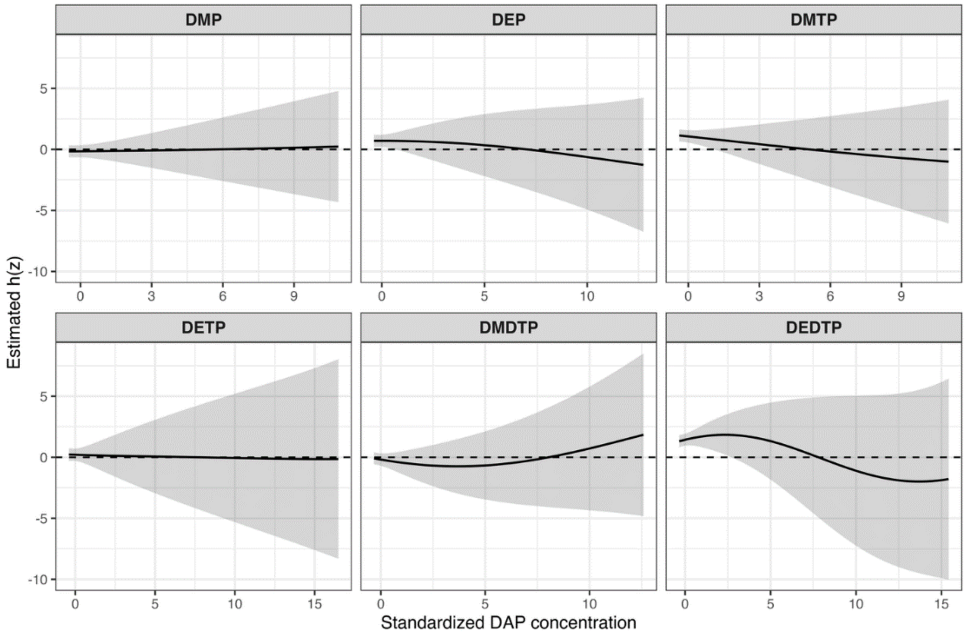

Figure S1. Univariate DAP exposure-response functions across epigenetic clocks. Panels show A (HorvathAge), B (HannumAge), C (PhenoAge), D (GrimAge), and E (GrimAge2). DAP—dialkylphosphate, DEP—diethylphosphate, DMP—dimethylphosphate, DMTP—dimethylthiophosphate, DETP—diethylthiophosphate, DMDTP—dimethyldithiophosphate, DEDTP—diethyldithiophosphate.

Figure S2A. Bivariate DAP Exposure–Response: HorvathAge

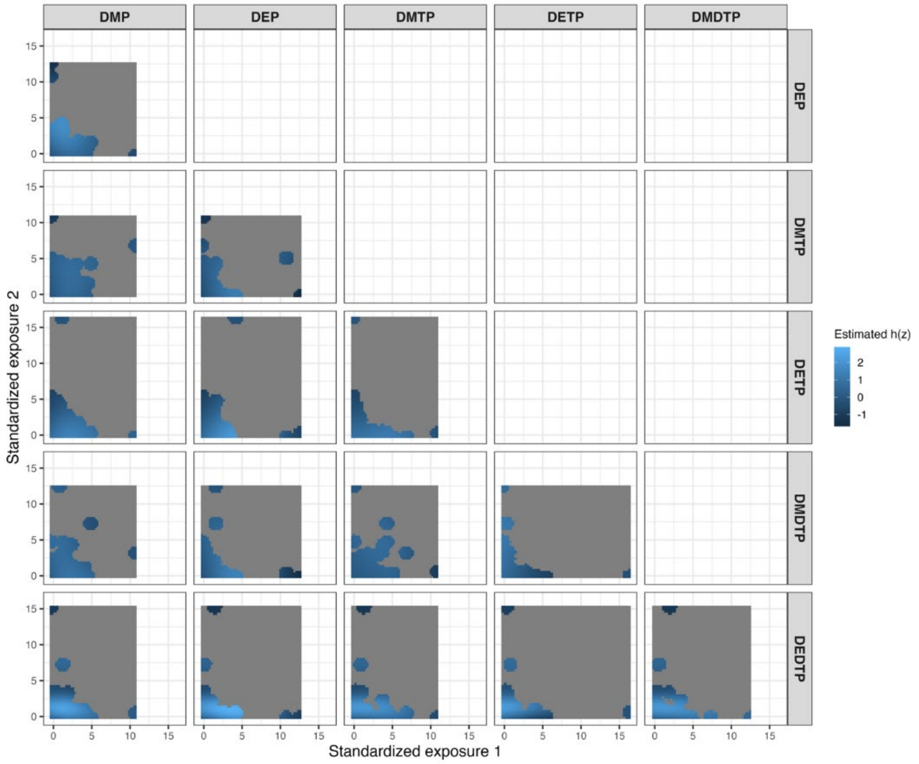

Figure S2B. Bivariate DAP Exposure–Response: HannumAge

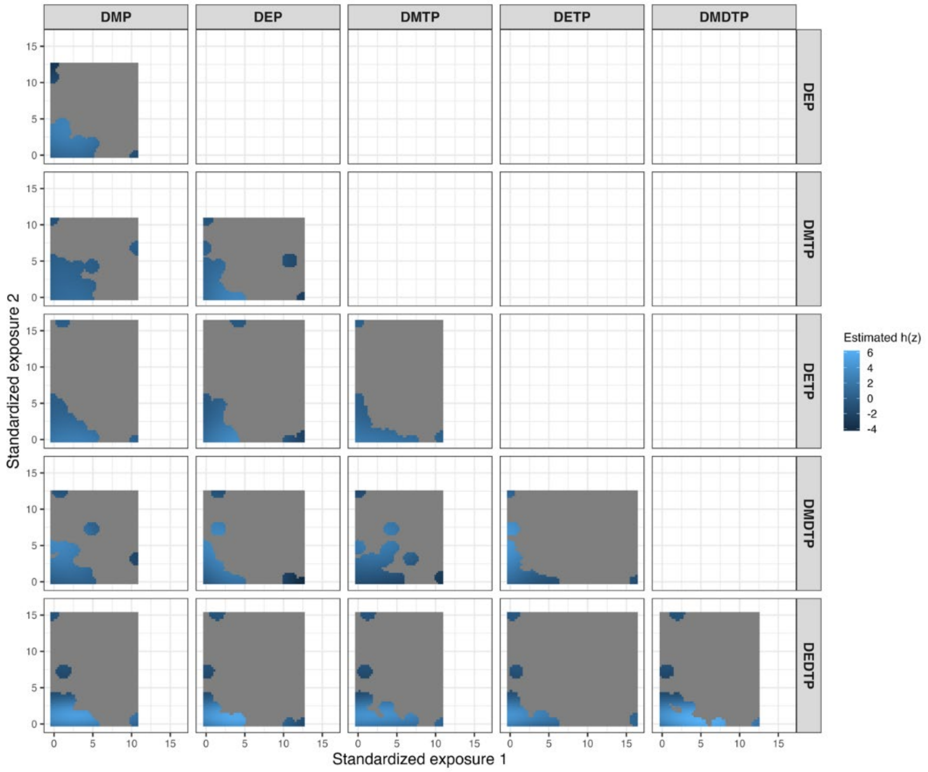

Figure S2C. Bivariate DAP Exposure–Response: PhenoAge

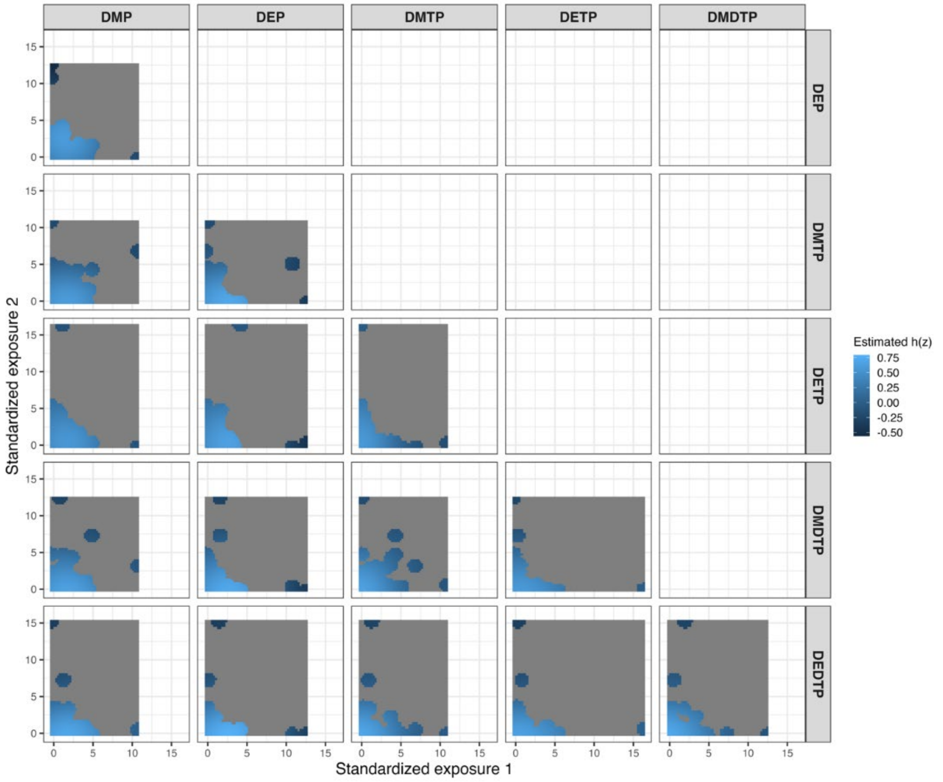

Figure S2D. Bivariate DAP Exposure–Response: GrimAge

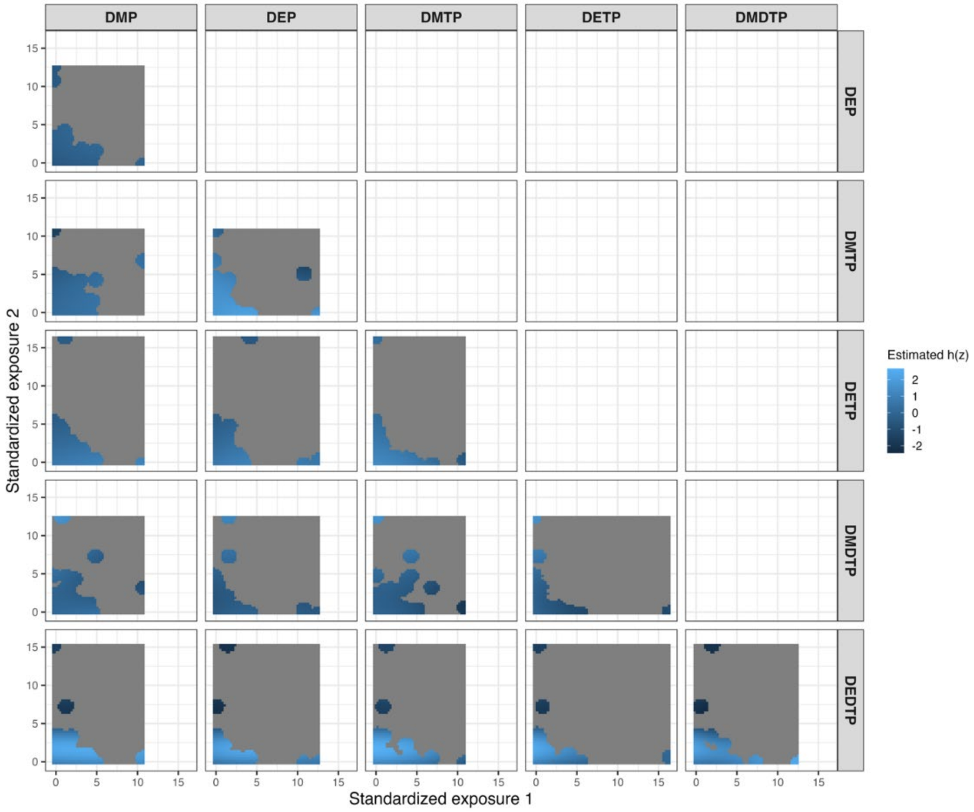

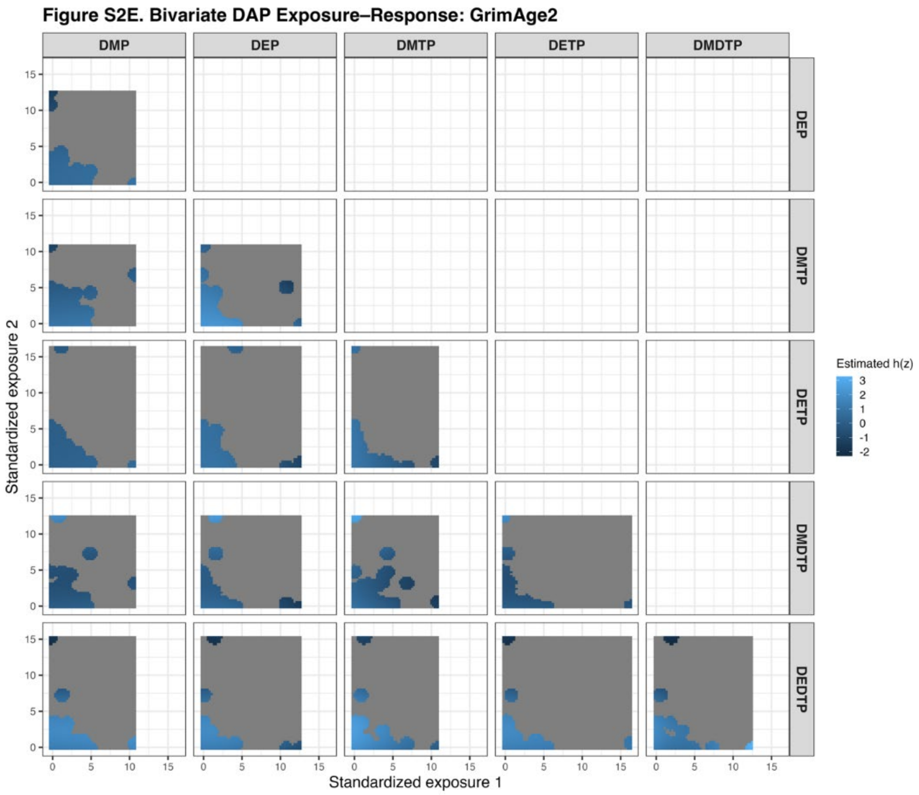

Figure S2. Bivariate DAP exposure-response functions across epigenetic clocks. Panels show A (HorvathAge), B (HannumAge), C (PhenoAge), D (GrimAge), and E (GrimAge2). Cell shading gives the posterior mean estimated exposure-response surface h(z) on the scale shown in each panel legend, with darker shades indicating lower and lighter shades higher estimated epigenetic age acceleration. Gray regions are combinations of the two standardized exposures that lie farther than one unit from any observed data point and are masked because the surface is not supported by data there. Axes are on the standardized (z-score) exposure scale; because the DAP distributions are strongly right-skewed, with a large proportion of values at the limit of detection, the standardized values extend well above zero and only slightly below it. DAP—dialkylphosphate, DEP—diethylphosphate, DMP—dimethylphosphate, DMTP—dimethylthiophosphate, DETP—diethylthiophosphate, DMDTP—dimethyldithiophosphate, DEDTP—diethyldithiophosphate.

Figure S3A. Pairwise DAP Interaction Functions: HorvathAge

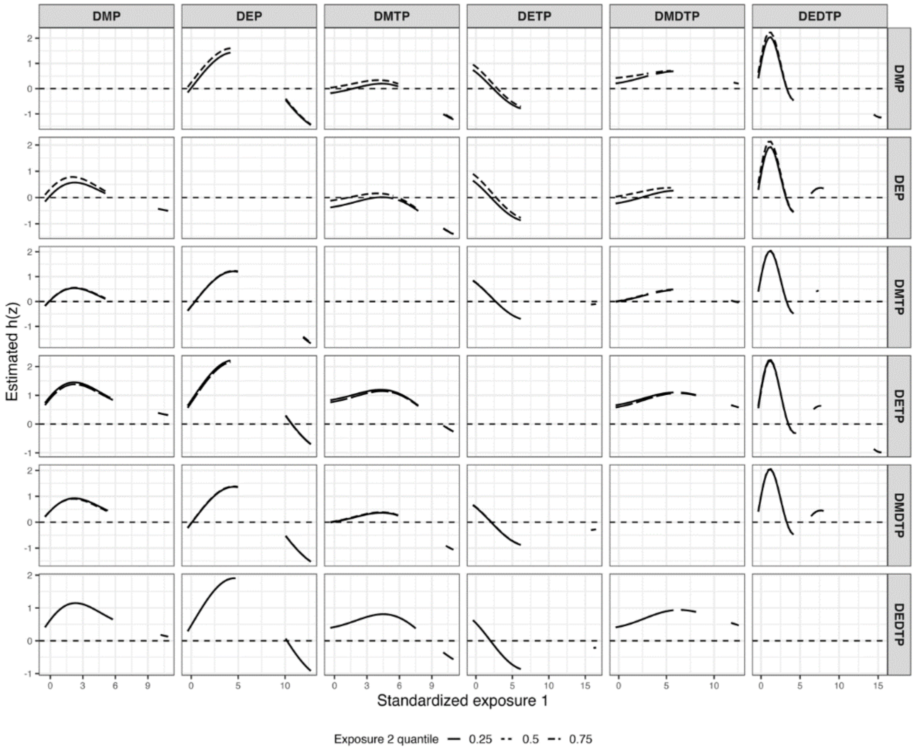

Figure S3B. Pairwise DAP Interaction Functions: HannumAge

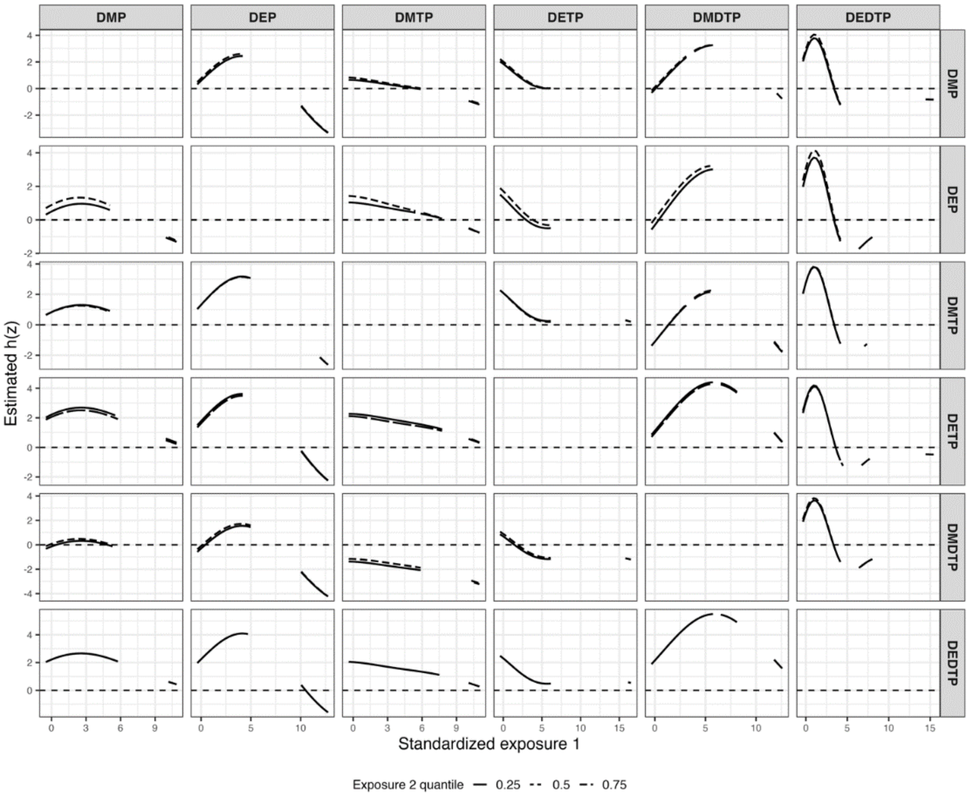

Figure S3C. Pairwise DAP Interaction Functions: PhenoAge

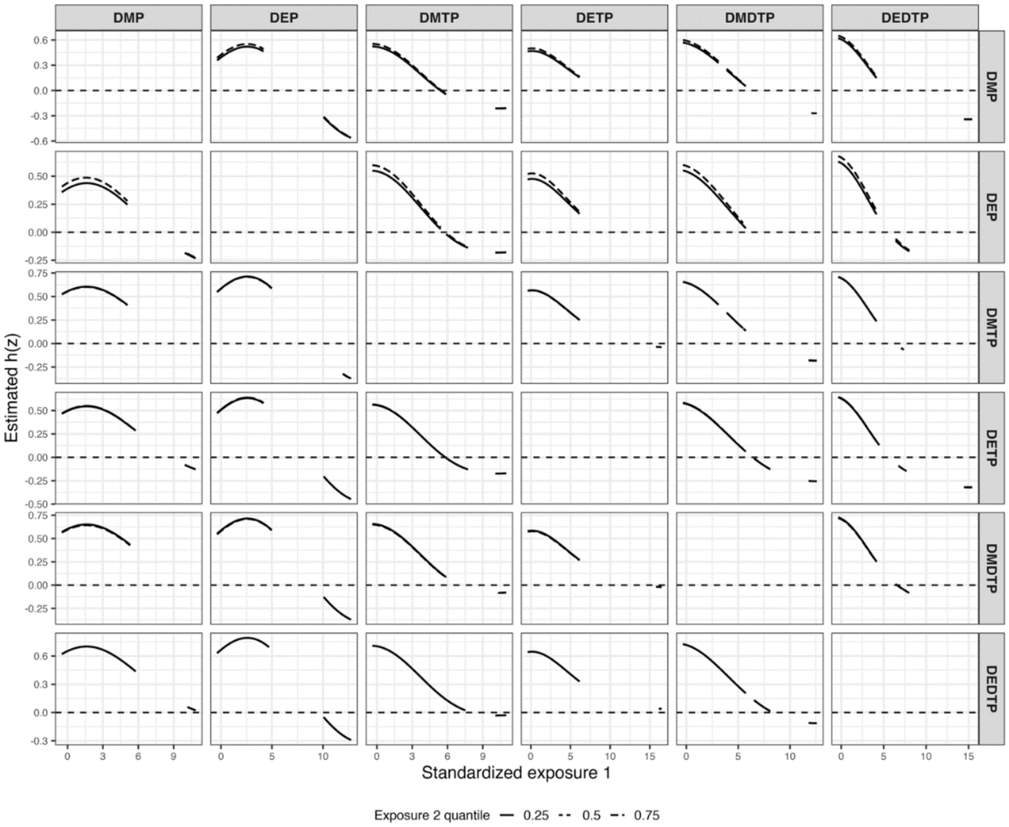

Figure S3D. Pairwise DAP Interaction Functions: GrimAge

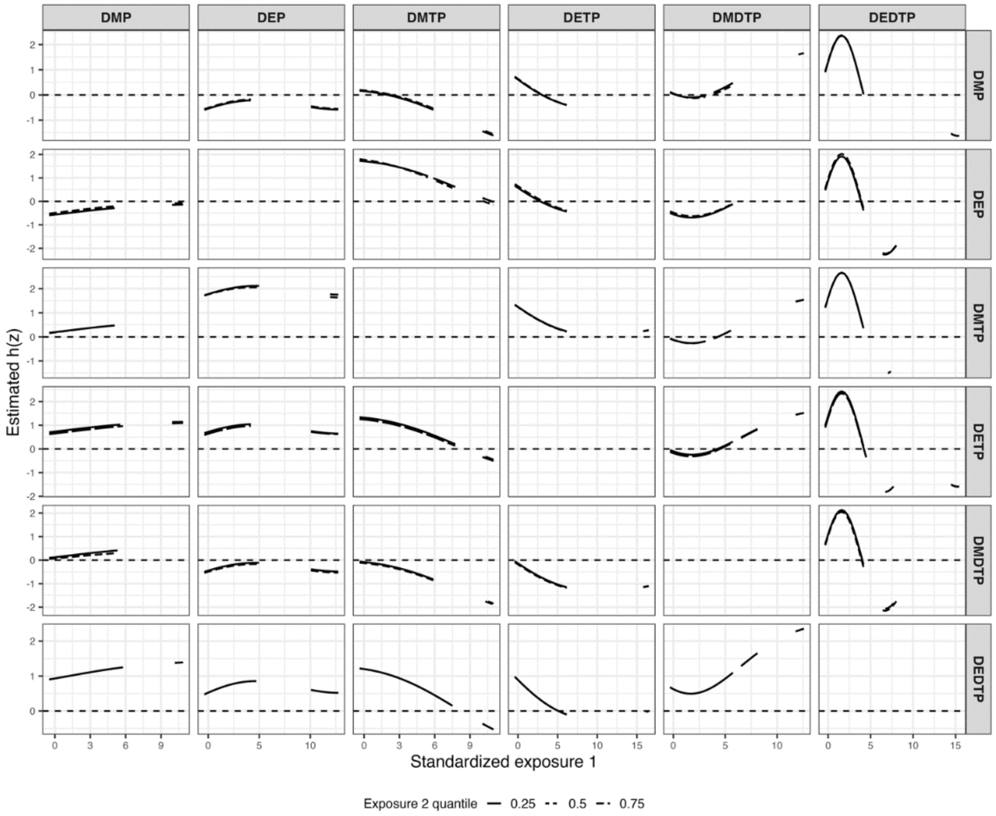

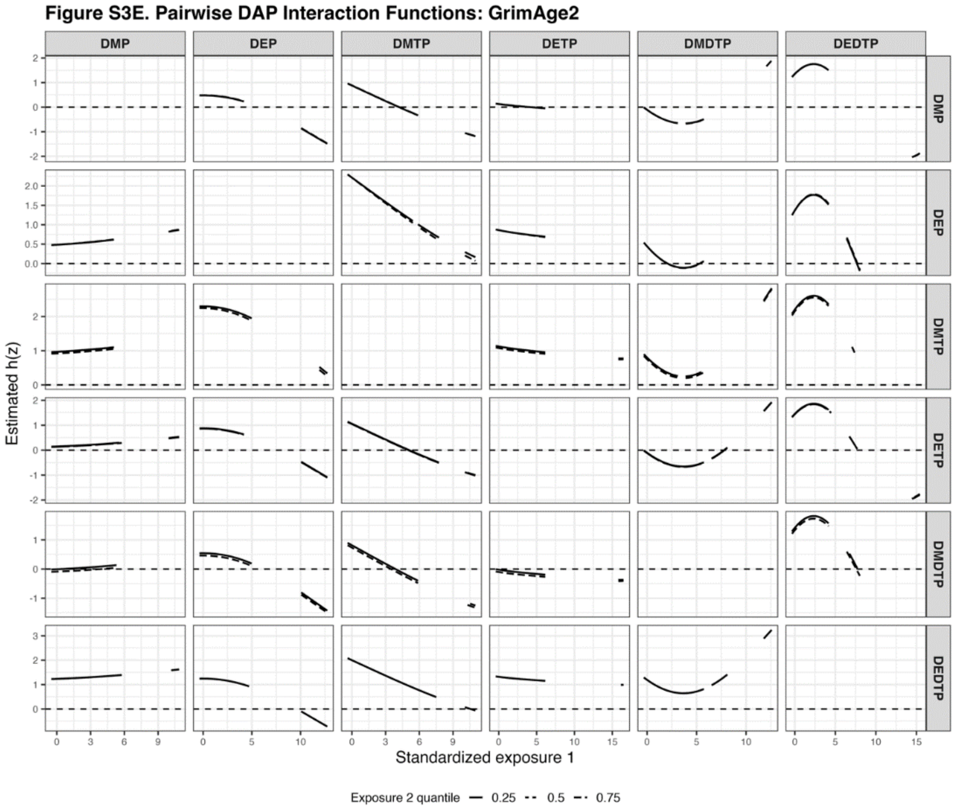

Figure S3. Pairwise DAP interaction functions across epigenetic clocks. Within each panel, the second exposure is fixed at its 25th percentile (solid line), 50th percentile (dashed line), or 75th percentile (dot-dashed line). Panels show A (HorvathAge), B (HannumAge), C (PhenoAge), D (GrimAge), and E (GrimAge2). DAP—dialkylphosphate, DEP—diethylphosphate, DMP—dimethylphosphate, DMTP—dimethylthiophosphate, DETP—diethylthiophosphate, DMDTP—dimethyldithiophosphate, DEDTP—diethyldithiophosphate.

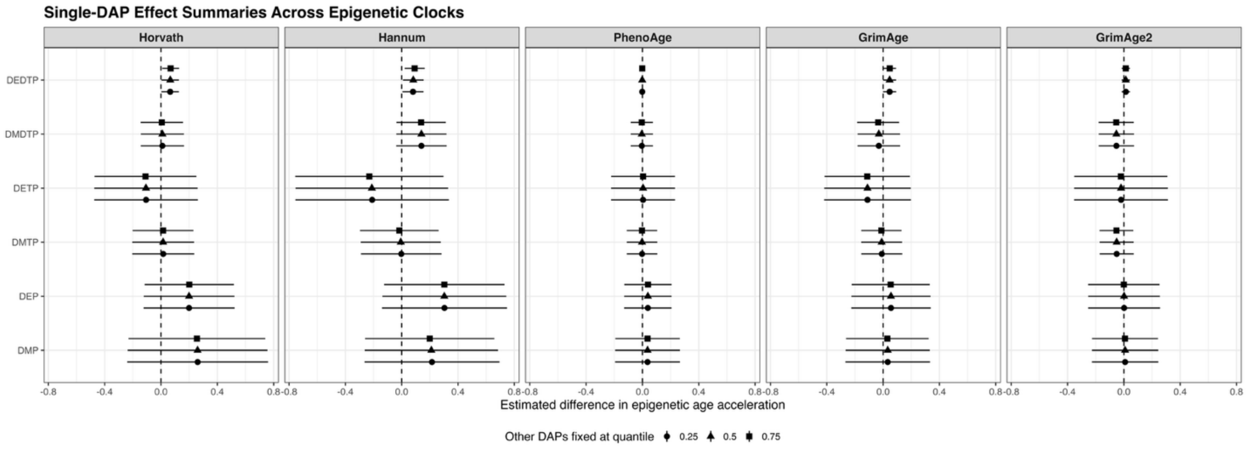

Figure S4. Single-DAP effect summaries across epigenetic clocks. Points show the estimated difference in epigenetic age acceleration for a change in the indicated metabolite from its 25th to its 75th percentile, with the remaining five DAPs held at their 25th, 50th, or 75th percentile; horizontal bars are 95% credible intervals. Panels are ordered HorvathAge, HannumAge, PhenoAge, GrimAge, GrimAge2; the panel labels Horvath and Hannum denote HorvathAge and HannumAge. Numerical posterior summaries for these contrasts are not tabulated, and the figure is intended to convey direction and relative precision rather than to support interval-based inference. (DEP—diethylphosphate, DMP—dimethylphosphate, DMTP—dimethylthiophosphate, DETP—diethylthiophosphate, DMDTP—dimethyldithiophosphate, DEDTP—diethyldithiophosphate).

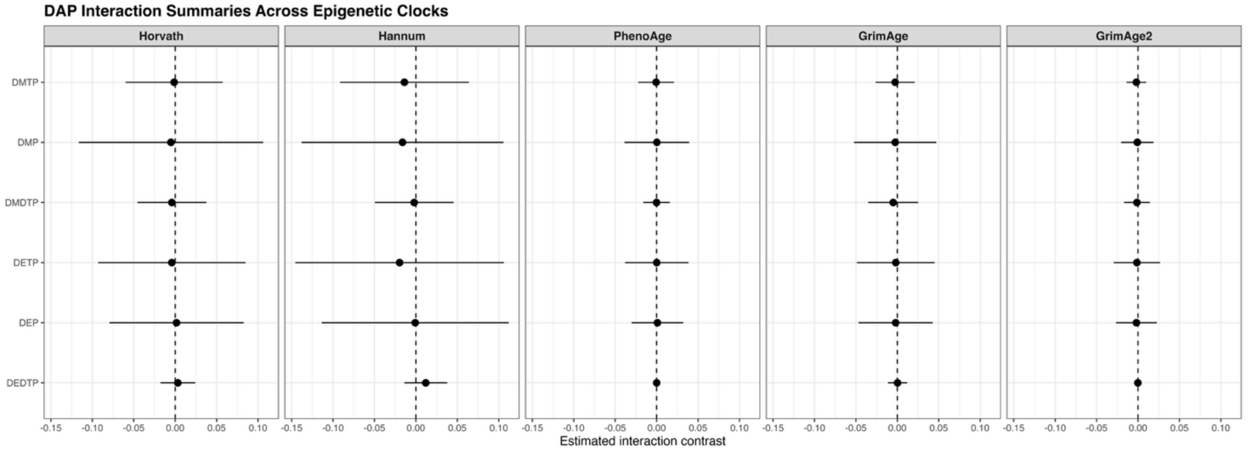

Figure S5. DAP interaction summaries across epigenetic clocks. DEP—diethylphosphate, DMP—dimethylphosphate, DMTP—dimethylthiophosphate, DETP—diethylthiophosphate, DMDTP—dimethyldithiophosphate, DEDTP—diethyldithiophosphate.

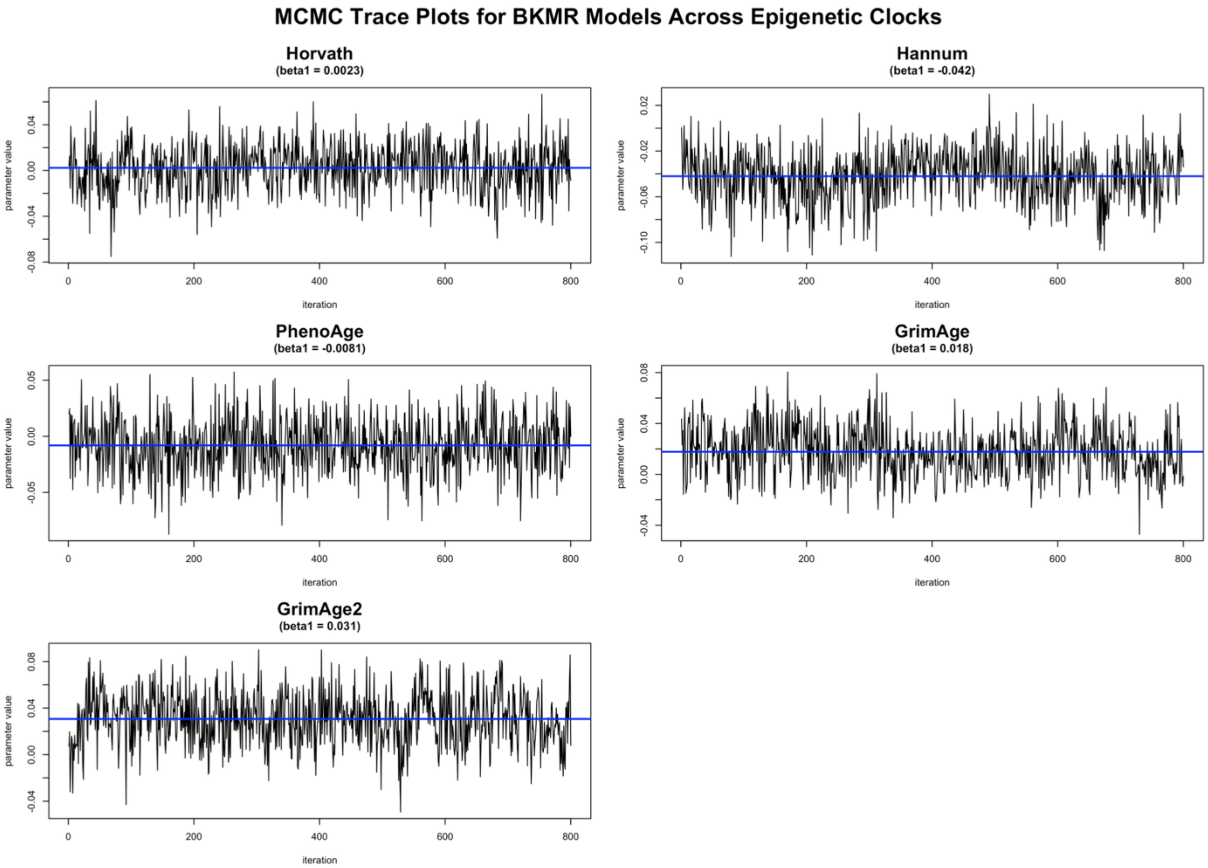

Figure S6. MCMC trace plots for primary BKMR models across epigenetic clocks. Each panel shows the sampled values of the first covariate coefficient (β1) from the 5,000-iteration model for that clock. The horizontal axis indexes plotted draws after thinning for display (every 5th draw after iteration 1,000), not raw MCMC iterations; the blue line marks the posterior mean. β1 is the coefficient for chronological age and is shown as representative of sampling behavior; convergence was additionally assessed by comparing posterior inclusion probabilities and overall mixture-response summaries across chain lengths and random seeds (Methods S1, Table S2).

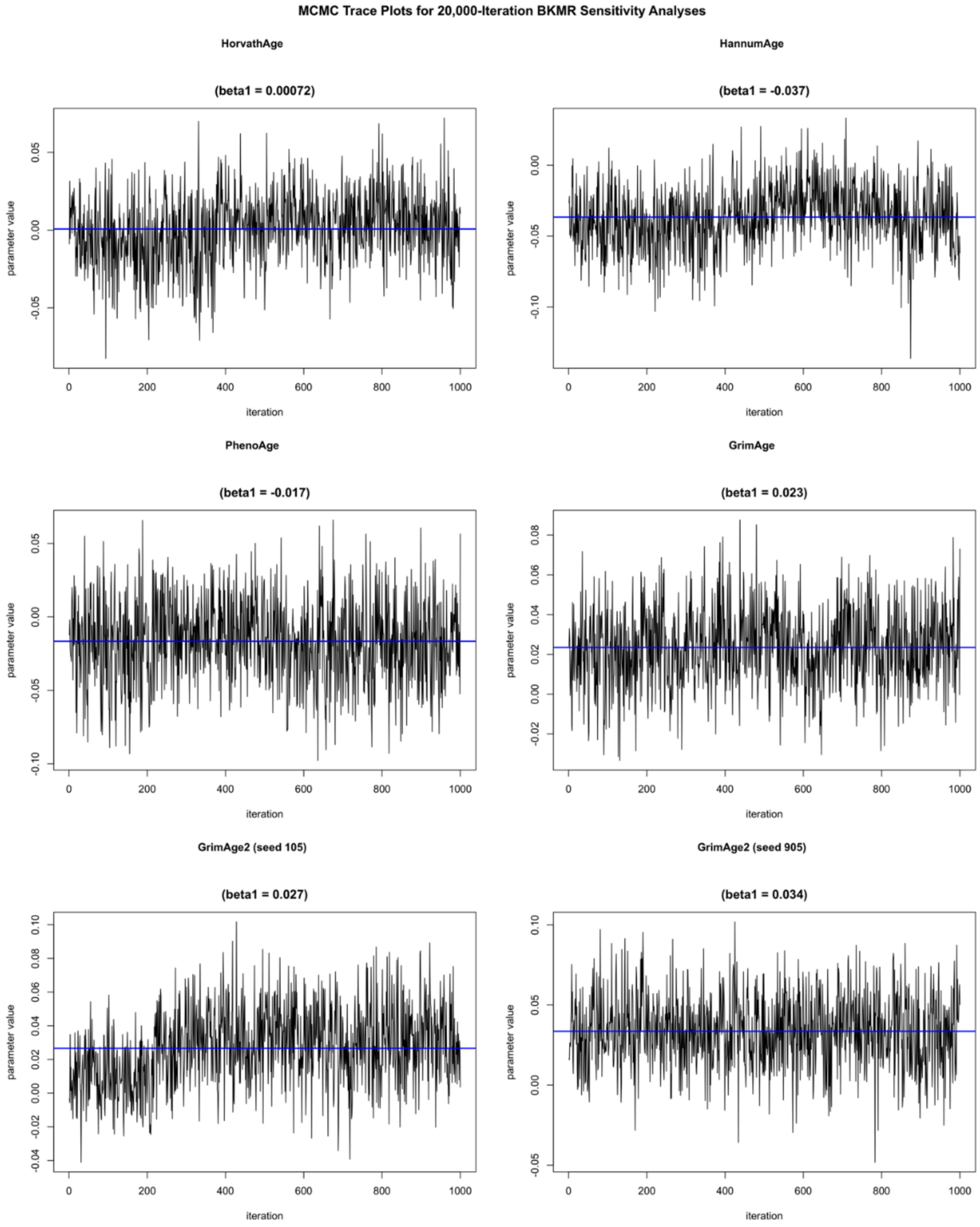

Figure S7. MCMC trace plots for sensitivity BKMR models across epigenetic clocks. Each panel shows the sampled values of the first covariate coefficient (β1) from the 20,000-iteration model for that clock. The horizontal axis indexes plotted draws after thinning for display (every 10th draw from iterations 10,001 to 20,000), not raw MCMC iterations. Six panels are shown: one 20,000-iteration sensitivity model for each of the five epigenetic clocks, plus an additional independent GrimAge2 model initialized with random seed 905. β1 is the coefficient for chronological age; the blue line marks the posterior mean.
